# Blood-Informative Transcripts Define Nine Common Axes of Peripheral Blood Gene Expression

**DOI:** 10.1371/journal.pgen.1003362

**Published:** 2013-03-14

**Authors:** Marcela Preininger, Dalia Arafat, Jinhee Kim, Artika P. Nath, Youssef Idaghdour, Kenneth L. Brigham, Greg Gibson

**Affiliations:** 1 Center for Integrative Genomics, School of Biology, Georgia Institute of Technology, Atlanta, Georgia, United States of America; 2 Saint Justine Children's Hospital, University of Montreal, Montreal, Quebec, Canada; 3 Center for Health Discovery and Well Being, Emory University Midtown Hospital, Atlanta, Georgia, United States of America; University of Chicago, United States of America

## Abstract

We describe a novel approach to capturing the covariance structure of peripheral blood gene expression that relies on the identification of highly conserved Axes of variation. Starting with a comparison of microarray transcriptome profiles for a new dataset of 189 healthy adult participants in the Emory-Georgia Tech Center for Health Discovery and Well-Being (CHDWB) cohort, with a previously published study of 208 adult Moroccans, we identify nine Axes each with between 99 and 1,028 strongly co-regulated transcripts in common. Each axis is enriched for gene ontology categories related to sub-classes of blood and immune function, including T-cell and B-cell physiology and innate, adaptive, and anti-viral responses. Conservation of the Axes is demonstrated in each of five additional population-based gene expression profiling studies, one of which is robustly associated with Body Mass Index in the CHDWB as well as Finnish and Australian cohorts. Furthermore, ten tightly co-regulated genes can be used to define each Axis as “Blood Informative Transcripts” (BITs), generating scores that define an individual with respect to the represented immune activity and blood physiology. We show that environmental factors, including lifestyle differences in Morocco and infection leading to active or latent tuberculosis, significantly impact specific axes, but that there is also significant heritability for the Axis scores. In the context of personalized medicine, reanalysis of the longitudinal profile of one individual during and after infection with two respiratory viruses demonstrates that specific axes also characterize clinical incidents. This mode of analysis suggests the view that, rather than unique subsets of genes marking each class of disease, differential expression reflects movement along the major normal Axes in response to environmental and genetic stimuli.

## Author Summary

Gene expression profiling of human tissues typically reveals a complex structure of co-regulation of gene expression that has yet to be explored with regard to the genetic and environmental sources of covariance or its implications for quantitative and clinical traits. Here we show that peripheral blood samples from multiple studies can be described by nine common axes of variation that collectively explain up to one half of all transcriptional variance in blood. Specific axes diverge according to environmental variables such as lifestyle and infectious disease exposure, but a strong genetic component to axis regulation is also inferred. As few as 10 “blood-informative transcripts” (BITs) can be used to define each axis and potentially classify individuals with respect to multiple aspects of their blood and immune function. The analysis of longitudinal profiles of one individual shows how these change relative to clinical shifts in metabolic profile following viral infection. The notion that gene expression diverges along genetic paths of least resistance defined by these axes has important implications for interpreting differential expression in case-control studies of disease.

## Introduction

The promise of personalized medicine is predicated on the assumption that people differ with respect to their disease susceptibility. To date, medical genomics has contributed primarily by defining genotypic risk based on common variants associated with disease and rare mutations predicted to disrupt proteins [1], [2]. To some extent this reflects the preconception that most individuals are normal with respect to disease predisposition, but that they harbor an excess of variants that increase risk for just a handful of different conditions. An alternative view is that normal variation is actually highly structured such that each person can be classified with respect to their position along a small number axes that define their susceptibility for metabolic, immune, pulmonary and psychological conditions in general. Systems genomics stands to contribute to personalized medicine if it can help to define these common axes of variation [3].

A promising approach is gene expression profiling. Most of the focus of GWAS for gene expression has been on local regulatory polymorphisms that explain over a quarter of the variance of individual transcripts [4], [5]. However, this approach has informed little about the genetic basis for the extensive and pervasive co-regulation of transcript abundance. Principle component and related analyses, including independent components [6] and non-negative matrix factorization [7], consistently reveal several dozen axes of variation that each explain a few percent of the total transcriptional variance, involving from 50 to 500 genes. Similarly, hierarchical clustering methods such as weighted gene co-expression network analysis (WGCNA [8]) and modulated modularity clustering (MMC [9]) partition the transcriptome into discrete modules of clearly co-regulated genes. These components or modules often seem to correlate with biological features of interest [10], [11]. For the most part the mechanisms responsible for the covariance are not understood.

A major problem with such methods is that they have relatively low repeatability, and hence it is not clear that they truly reflect common regulatory mechanisms. The first principal component of whole blood gene expression is highly conserved across multiple studies that we have conducted, in that the loadings of thousands of transcripts significantly correlated with PC1 are similar, although a fraction of the transcriptome seems susceptible to methodological differences (Figure S1A). The next several PC also capture similar patterns of covariance, but they are not the same between studies (Figure S1B) and change substantively as a result of normalization procedures. Thus, the eigenvectors of each transcript with PC3 in one study or analytical approach, may be correlated with PC3 and PC4 in another. While advanced factorization procedures may resolve some of these issues, these components of covariance are to some extent contingent on latent features specific to each dataset, and alternative approaches are needed to discover common axes of variation.

One such strategy was adopted by Chaussabel et al [12], who queried multiple peripheral blood gene expression datasets for conserved modules of transcripts that differ according to disease state. They described 28 modules and showed that the severity of systemic lupus erythromatosis corresponded to up-regulation of several of the modules, while transplant and melanoma patients showed other patterns of differential modular expression. Here ask whether the modules are also present in healthy adults, and explore some implications of the observation that they indeed are conserved, with the following work flow. Principal component analysis of just the genes in each of the 28 modules indicates that their covariance is highly conserved in two healthy adult studies, but that they can be collapsed into 6 meta-modules, and we also identify 3 additional co-varying gene sets. We then show by multiple linear regression that of the order of one half of the transcriptome is highly significantly correlated with one or more of these meta-modules, which we redefine as 9 Axes of covariance. Ten of the genes that are most closely associated with each Axis are selected, and these are also found to be highly co-regulated in seven other peripheral blood transcriptome studies where they show significant correlations with genetic, environmental, or phenotypic variables. Finally, we demonstrate that these 90 Blood Informative Transcripts have the potential to track changes in immunological and metabolic status, and argue that they may thus be used to evaluate pre-clinical risk of disease.

## Results

### Defining 9 Common Axes of Peripheral Blood Variation

**Figure 1:**
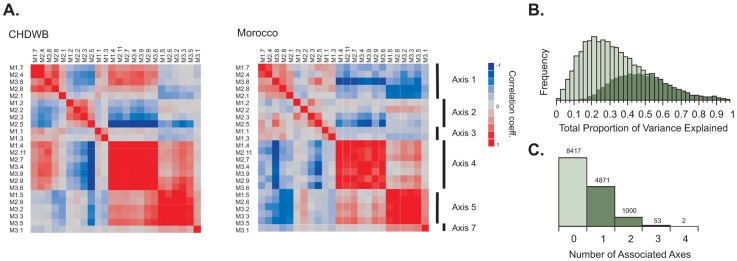
Common axes explain a large proportion of expression variation. (A) Hierarchical clustering of the PC1 scores for 24 Expression Modules in Ref [12] in the Atlanta CHDWB and Morocco datasets shows complete agreement in clustering into 6 meta-modules. These define 6 of the Axes described here, while a 7^th^ Axis emerged on further decomposition of Module 3.1 (B) The frequency distribution of proportion of variance explained by all 9 Axes for each of 14,343 transcript probes (light green) and 7,538 transcript probes (dark green) Bonferroni significant for at least one Axis in a multiple regression. Inclusion of the two additional axes not corresponding to the Chaussabel modules only explains an extra 4% of the variance relative to the first seven. (C) The number of Bonferroni significant axes per transcript in the CHDWB dataset, showing that 39% (5622/14343) of transcript probes associate most strongly with a single axis.

In order to assess whether transcripts in each of the 28 modules in [12] are also co-expressed in healthy adults, we generated the first principal component scores for each module in 189 individuals in a predictive health study in Atlanta (the CHDWB study, first described here), and in 208 Moroccans [13], using transcript abundance measures from Illumina HT12 microarrays. In general, over one quarter of the variance in each Chaussabel module was captured by its first principal component (Table S4), in both studies, implying significant co-regulation, as expected. However, the PC1 scores were themselves highly correlated between modules, with a very similar structure to which modules co-vary in the two studies, suggesting that there may be a smaller number of conserved axes in healthy adults, subsets of which diverge among disease classes. Based on the clusters evident in Figure 1A, we condensed them into six meta-modules, and also discovered three more sets of co-expressed genes, resulting in nine *Axes* of common peripheral blood gene expression variation (see Methods for details of the workflow). It is likely that there are other minor Axes, and some splitting into sub-Axes is possible, but an independent line of evidence described in Figure S2 supports the conclusion that these nine axes of variation are the major common and replicated sources of covariance in peripheral blood gene expression.

**Figure 2:**
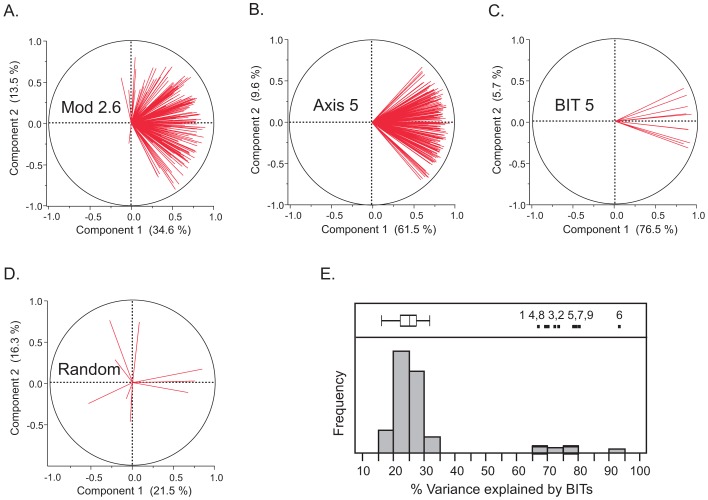
Covariance of gene expression in Modules, Axes, and BIT. The first four panels illustrate the extent of covariance of gene expression by plotting the loadings for the first two principal component axes for genes related to Axis 5 in the Morocco study in: (A) Module 2.6 identified by Chaussabel et al (ref 12; 167 probes for 105 genes); (B) Axis 5 (which derived partially from Module 2.6; 175 probes for 150 genes); (C) the Blood Informative Transcripts for Axis 5 (10 probes for 10 genes); and (D) a typical random sample of 10 probes. The percent variance explained by the first two components is indicated. Panel (E) shows a histogram of the percent variance explained by component 1 for 100 random sets of 10 probes, relative to the 10 BIT Axes for Morocco to the right (Axis number is in top portion of the panel). Eight of the 9 Axes are plotted for the Atlanta CHDWB dataset in Figure S3A.

The genes associated with each of these axes were then cross-matched between the CHDWB and Moroccan studies. Any transcript that was associated with the first PC of the corresponding meta-module at Bonferroni significance in a multiple regression, in both studies, is included in a list of between 99 and 1028 genes that define each axis (Dataset S1). In the CHDWB, the axes collectively capture 37% of total transcript abundance (the average R-squared for a multiple regression with all 9 axes over 14,343 probes), and 49% of the variance for the 53% of the peripheral blood transcriptome (7538 probes) that co-varies with at least one Axis at Bonferroni-corrected significance (Figure 1B). In Morocco, the explanatory power of the axes is even stronger, with 51% of the variance of all transcripts captured by the nine axes. We then identified the 10 transcripts with the highest correlation within each axis, in both studies, that are not correlated with any of the other axes. These 10 transcripts can be regarded as Blood-Informative Transcripts (BITs: Table S1) and the PC1 score in turn derived from these BITs represent Axes scores that potentially define an individual's immune status. The process of capturing variation with modules, then Axes and BIT is illustrated in Figure 2, which also shows (panel E) how the variance explained is markedly greater than random subsets of genes. Averaged across both studies and all 9 Axes, 79% of the variance of each set of 10 BIT transcripts is captured by their Axis scores. Note that, unlike principal components of the entire transcriptome, the nine Axis scores are not orthogonal, reflecting residual covariance even among the Axes. The axes appear to have replicated effects transcriptome wide, since the estimated regression coefficients for each BIT transcript on each Axis score are highly conserved between Atlanta and Morocco (Table S2 and Figure S3B).

We then confirmed that the Axes are not just a feature of these two studies by interrogating five other adult peripheral blood-derived microarray datasets: our own published study of 100 adults in Brisbane Australia [14], and four studies conducted by other groups, one of tuberculosis and other infectious diseases in Africa and England [15], a study of celiac disease [16], the DILGOM metabolic disease study in Finland [17], and a twin study of both peripheral blood and lymphoblast cell lines in Brisbane [18]. These studies each adopted different procedures for peripheral blood extraction, including Tempus and Paxgene isolation of whole blood, as well as leukocyte or peripheral blood monocyte isolation. In each case the BIT are much more strongly correlated than are random permutations of 10 genes, and the BIT Axis score captures as much variance as in the Atlanta study, with particularly strong conservation of Axis 6 (Table S2). Transcripts in Axes 4, 9 and 10 have the weakest correlation with one another, although each of these still include several hundred genes. Notably, the covariance between the Axes is somewhat study-specific as shown in Figure S4. Splitting each study into halves preserves the overall covariance pattern within each study, but most of the pairwise axis correlations are significantly different between studies. This mainly reflects a combination of technical differences (platform, source of blood, statistical methods) rather than biological ones since as few as 25 samples can recover the study-specific pattern. Nevertheless, there are many consistencies, such as the positive correlations between Axes 1 and 3, and between Axes 5 and 7, or the negative correlation between Axes 1 and 5, and between Axes 2 and 4 (see also Figure S5 for comparison across six studies).

### Biological Function of the Axes

Enrichment for biological functions associated with each Axis was assessed using the ToppGene suite [19] to perform gene set enrichment analysis. Table 1 shows annotations that suggest involvement in T-cell signaling (Axis 1), B-cell signaling (Axis 3), cytokine-mediated signaling (Axis 5), and anti-viral response (Axis 7). Axis 4, like Axis 5, shows evidence for enrichment of genes involved in inflammation but the former with an emphasis on the macromolecular complexes. Axis 2 is not obviously associated with a sub-class of immune activity, but at least partially reflects erythrocyte and platelet function, as a small but significant number of transcripts are annotated to oxygen binding and coagulation. Axes 9 and 10 appear to reflect some coordination of receptor-mediated signaling and translational regulation in lymphocytes. Additionally, as indicated, these axes are enriched for genes identified as influencing abnormal mouse phenotypes, and in a few cases human diseases.

**Table 1:** Features of the Blood Expression Axes.

Axis	Gene Ontology^1^	Abnormal mouse phenotype^1^	TF/miR^1^^,^ 2	Human Disease^1^	N genes^3^
Axis 1	Translation	T cell physiology (4.0E-06)	ELK1	T-negative SCID	866
	Ribosome constituents	Leukopoiesis (4.0E-04)	NRF2	anemia	
Axis 2	Oxygen transporter activity	Platelet aggregation (9.0E-04)	GATA3	hemolytic anemia	237
	Wound healing	Haematopoiesis (8.0E-06)			
Axis 3	B-cell activation	B-cell morphology (4.0E-19)	miR-486	SLE	99
	External to plasma membrane	Immunoglobulin level (4.0E-16)	miR-146a	Immunodeficiency	
Axis 4	mRNA metabolism, RNA splicing	Embryonic lethality (3.0E-10)	ELK1, NRF1		982
	Intracellular transport	Low embryonic growth (5.0E-06)	miR-590, 548, 561		
Axis 5	Cytokine receptor activity	Adaptive immunity (7.0E-14)	ETS2, PEA3, AP1		1028
	Inflammatory response	Innate immunity (3.0E-14)	miR-1207	Liver disease	
Axis 6			miR-483, 590		550
Axis 7	Viral response	Response to infection (4.0E-13)	IRF, ISRE, ICSBP		169
	Interferon-mediated signaling	Susceptibility to viral infection (3.0E-12)			
Axis 8	RNA processing	B cell activation (0.03)	NRF1, ARNT		571
			miR-590, 548, 607		
Axis 9	Signal transduction by phosphorylation	B cell number (7.0E-05)	STAT5A, STAT6		242
	Programmed cell death	T-cell morphology (2.0E-04)	miR-548, 155, 34b, 603, 103		

1 See Dataset S3 for details of the GO catergories, number of genes, and significance levels.

2 Simply reflecting enrichment frm the ToppFun analysis, not meant to imply that these are the only regulatory factors or that there is direct evidence for their involvement in regulation of the axes. Dataset S3 shows that each bind only a subset of genes in the axis.

3 The number of genes associated with each Axis in a multivariate model including all 9 Axes, at Bonferroni significance and in common between the CHDWB and Morocco studies.

Three lines of evidence indicate that these functional enrichments are not solely due to differential abundance of the respective cell types. First, the correlation between relative cellular abundance and Axis scores was explored in the CHDWB study, where neutrophil, lymphocyte, monocyte, platelet, and red blood cell counts were available for each individual from the same blood sample used to generate the RNA, is generally modest and insignificant. Two exceptions are Axis 5 with neutrophil counts, accounting for 34% of the axis variance, and Axis 1 with T cell counts, largely due to a small fraction of outliers (Figure S6). The ratio of neutrophils to lymphocytes is also highly correlated with Axis 5. Second, fitting cell counts during normalization of the gene expression data to remove the effect of cellular abundance does not affect the existence of the Axes of variation. Although the degree of correlation of genes that define the axes changes, all 9 Axes continue to be observed, and the BITs tend to retain their covariance with one another. Third, as noted above, the axes are observed across diverse blood sample types including leukocyte-enriched, PaxGene and Tempus whole blood preparations, and Ficoll-gradient purified PBMC. We also find very strong retention of Axes 4, 5 and 6 in neutrophil preparations from a cystic fibrosis cohort (in preparation).

It is noteworthy that Axis 2 is not associated with platelet or erythrocyte count (Rsq<0.01, p>0.25 in both cases), despite being enriched for platelet and red blood cell functions. We also asked whether transcripts known to be enriched in these two cell types are over-represented in Axis 2 genes, but they are not, from which we conclude that variation in Axis 2 is not due to variation in the abundance of these two anuclear cell types. It is possible that other sub-classes of immune cells contribute to the observed variation (for example, CD4 and CD8 subsets, although these two genes are positively correlated for all 5 probes in both studies and are not strongly associated with the Axes). Neutrophils, monocytes and lymphocytes are the predominant white blood cells by number in peripheral blood, so more minor cell types constituting less than 5% of the cells (dendritic cells, for example, but not eosinophils which we also measured) may also contribute, though it is mathematically unlikely for them to account for the fold changes at either end of the axis distributions, especially for hundreds of genes. Nevertheless, the pervasiveness of the axis associations and the impact of sampling method on the relationship between the axes documented in Figure S4 would be consistent with some contribution of cellular composition to the variance captured by the axes. It is also feasible that factors that increase abundance of each cell type, for example neutrophils, also impact expression within the individual cell types.

### Genetic and Environmental Regulation of the Axes

The existence of inter-individual variation in gene expression along defined axes must reflect the activity of regulatory networks that have evolved to ensure that thousands of transcripts co-vary in a conserved manner. We present here evidence for both genetic and environmental influences, and provide the relevant data in Dataset S2.

**Figure 3:**
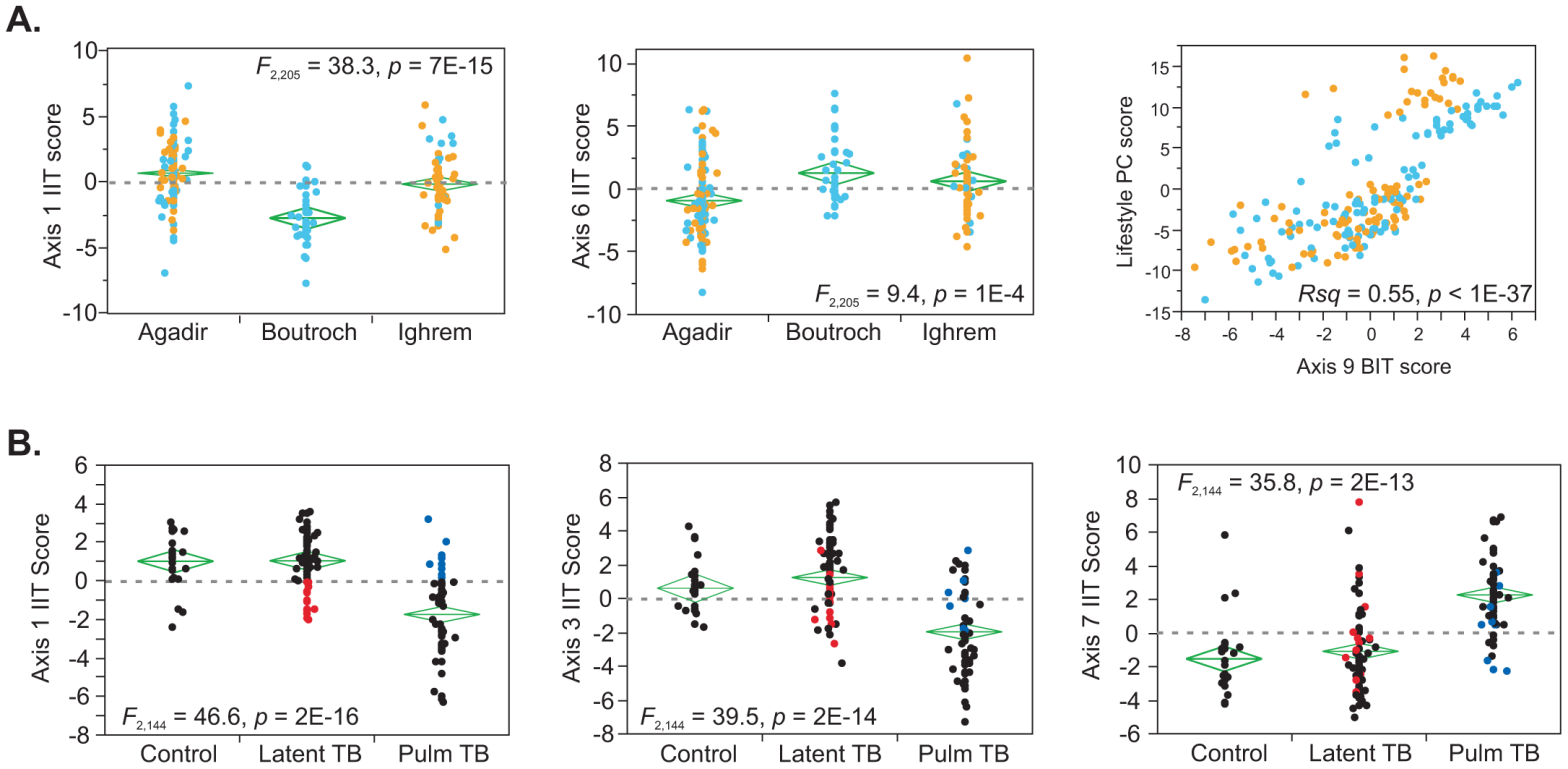
Environmental influences on the Axes. (A) Differences in Axis scores between geographic locations in Morocco (City of Agadir, villages of Boutroch and Ighrem: Berbers, blue; Arabs, yellow) are restricted to Axes 1, 2, 6, and 9, where Axis 6 distinguishes rural from urban, and Axes 1 and 2 distinguish Boutroch only. A grouping of Boutroch residents with Arab women in Ighrem against all others, which represents the major “lifestyle” effect on gene expression [13] generates a PC score that is highly concordant with Axis 9. (B) In the study by Berry et al [15], pulmonary tuberculosis differs from latent tuberculosis and healthy controls along four Axes (1, 3, 5 and 7), but not along Axes 2 or 5. A small number of individuals shown in red and blue are mis-classified for Axis 1 and 3, and likely have intermediate TB activity status, but this is not apparent for the differentiation of the interferon-response axis 7. None of the axes distinguish latent TB from control. PC1 for the diagnostic transcripts reported in [12] is highly correlated with Axis 1 (London sample R squared = 0.73, p = 10^−28^, South African sample R squared = 0.81, p = 10^−19^) and likely reflects divergence along this axis.

In southern Morocco, expression of over a third of the peripheral blood transcriptome was previously shown to differ between residents of rural Berber villages and of the city of Agadir [13]. Figure 3A now shows that this differentiation is particularly notable for Axes 1, 6, and 9, and to a lesser extent Axis 2. In fact, the first principal component of the 200 genes most strongly differentiated by lifestyle is coincident with Axis 9 and includes the *SNORD* and nucleolar component enrichment previously noted [13]. Part of the geographical genomic effect is thus likely to affect exposure to environmental agents that impinge on axes that seem to relate to anti-microbial responsiveness. Whether these are biotic or abiotic remains to be assessed, but the considerable impact of lifestyle on peripheral blood profiles is clearly affirmed. The differential expression is not uniform across all genes, and is to some extent constrained along the existing regulatory axes we have identified.

Another likely class of environmental influence is infectious disease. Since the Axes were derived from modules that were previously shown to diverge between immunological conditions, we asked whether the Axes differ according to infection status in the tuberculosis study [15]. Figure 3B shows that this is indeed the case. The study includes individuals with latent and active pulmonary TB, as well as healthy controls and adults infected with staphylococcus or streptomyces. Specific Axes do differ significantly between these four infection states, to some extent reflecting prior expectation: as shown here, Axes 1, 3 and 7 differ significantly between individuals with active and pulmonary TB, and this is observed independently in the London and South African sub-samples. The first principal component of the 86 transcript signature of active TB described in [15] is essentially indistinguishable from our Axis 1. Interestingly, individuals with intermediate level scores of the lymphocyte-associated axes 1 and 3, have diverse scores for Axis 7, whose gene annotation associate it with anti-viral response. It is possible that viral co-infection affects the clinical course of TB progression, and our data would suggest some uncoupling of the two classes of pathogen response. Alternatively, Axis 7 may have a direct role in TB response.

**Figure 4:**
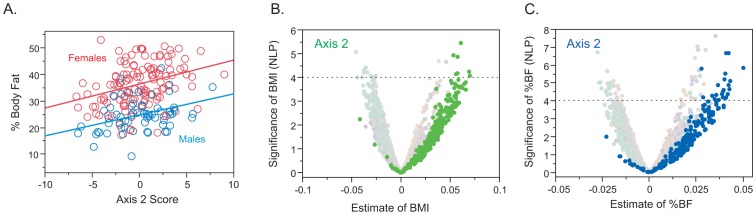
Relationship between BMI or Percent Body Fat (%BF) and Gene Expression in the CHDWB study. (A) Regression of percent body fat (%BF) on Axis 2 score in both sexes. (B) and (C) Volcano plots of significance against effect size for transcripts associated with Axes 2 with BMI (green) and with %BF (blue). The X axis is the estimated correlation between transcript abundance and the trait for each of 3913 unique genes in the 189 individuals in the cohort that are correlated with one or more axes in both Atlanta and Morocco. The dashed horizontal line is at p = 10^−4^: only 10 points are expected above this line, and these do not yield a clear enrichment for gene ontology classes, but the Axis analysis shows clear up-regulation of the two axes in general.

One other potential source of differentiation is overall health status. A complete description of the relationship between gene expression and clinical covariates in the CHDWB will be presented elsewhere, but we note that body mass index (BMI) and biomarkers of inflammation do correlate with specific Axes of peripheral blood gene expression (Figure 4). BMI is itself correlated with percent body fat, and both of these measures show a strong association with Axis 2 (R-squared = 0.07, p = 0.0004) and this is true in both males and females. The association of BMI with Axis 2 is replicated in our Brisbane Red Cross study [14] (R-squared = 0.11, p = 0.0014, Figure S7), and also in the DILGOM Finnish study [17] (R-squared = 0.074, p = 3.4×10^−8^; BMI data was not available for the Morocco study).

### Longitudinal Change in Axes with Clinical Status

**Figure 5:**
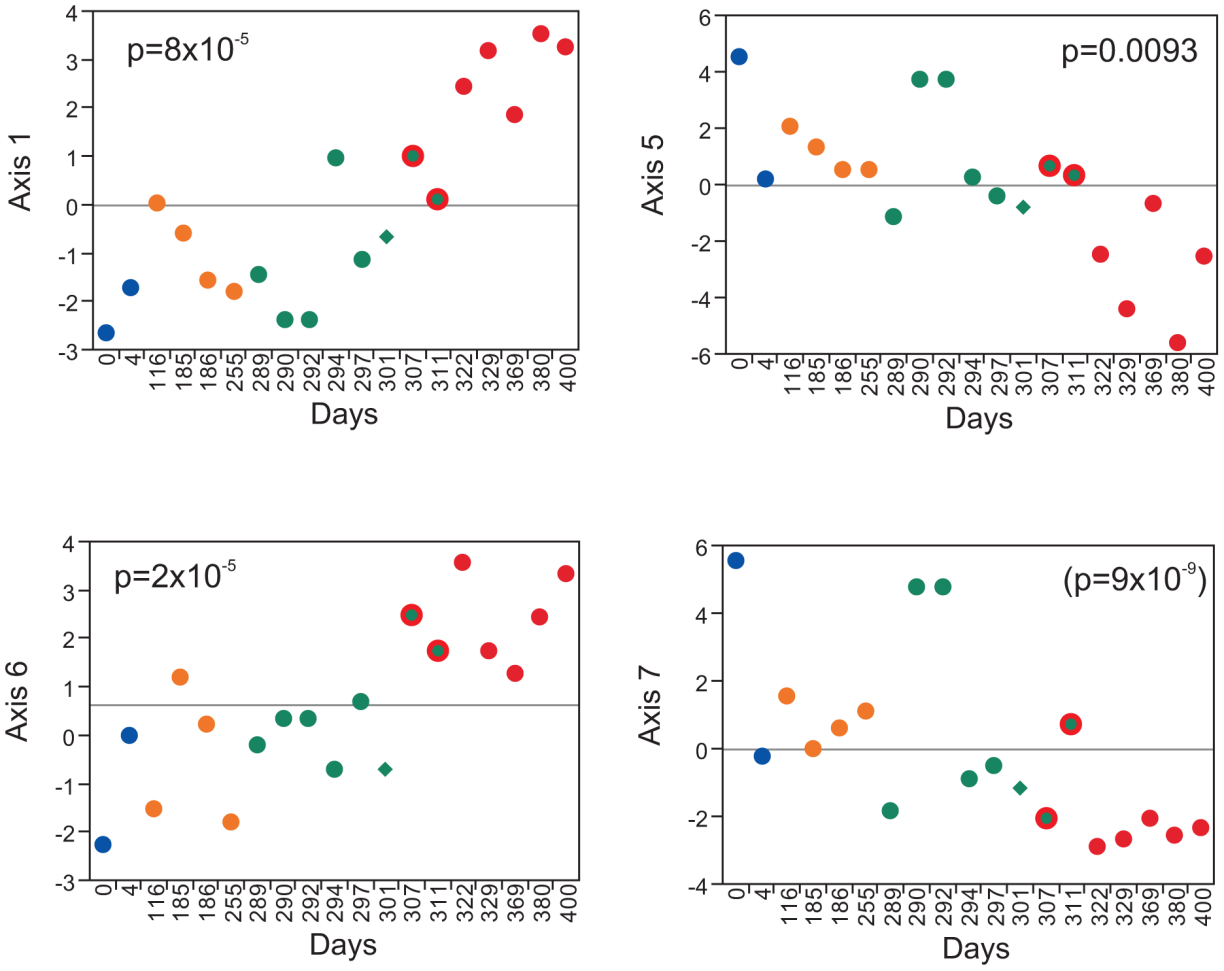
Axis analysis of the Snyderome. 19 sequential RNA-Seq profiles were mined for BIT Axis scores, which are plotted relative to sampling day for the individual described in [20]. P-values indicate the signifcance associated with t-test comparison of high blood glucose versus normal (Axes 1, 5 and 6) or the comparison of acute phase of viral infection - Days 0, 290 and 292 - versus the remainder. Colors indicate the phases of human rhinovirus infection (HRV; blue), recovery (yellow), respiratory syncitial virus infection (RSV; green), and pre-diabetes/high blood glucose (red). The green triangle corresponds to a spike in cytokine profile at day 301, which does not obviously impact the Axes. The RSV had not yet cleared at days 307 and 311 when the pre-diabetic state first became apparent, so these points are shown in green and red. The other Axes did not show significant changes.

In order to demonstrate the potential utility of the BIT axis scores for revealing changes in personal genomic response to disease, we assessed the axis scores derived from the BIT in the so-called “Snyderome” longitudinal RNA-Seq dataset [20]. Nineteen sequential whole blood gene expression samples were extracted from GEO accession GSE33029 for the individual who, over a period of 400 days, experienced two respiratory virus infections and clinical signs of diabetes onset. Figure 5 shows the Axis scores for the four axes that differ significantly between clinical phases. Both Axes 1 and 6 show highly significant elevation (t-test, p<10^−4^)during the high blood glucose phase (red points) with time of onset after day 301 (green diamond), apparently with the change occurring one or two weeks earlier for Axis 6, before the second viral infection (green points) had cleared. Axis 5, the inflammatory axis, shows the opposite trend, possibly simply reflecting negative covariance with Axis 1 which is strong in these 19 samples. Axis 7 is particularly noteworthy for the elevation (t-test, p<10^−8^) during the two to four day acute phase of both respiratory viral infections, as expected given the enrichment for interferon signaling and viral response genes. These changes to some extent mirror spikes and trends in the autocorrelation analysis reported in [20], and are consistent with the reported modification of T cell signaling, and insulin response pathways. Unfortunately, RNA profiles were not obtained for the time period subsequent to return to normal blood glucose levels, precluding assessment of whether the BIT can also be considered biomarkers for an individual's return to good health.

## Discussion

There are three aspects of the axes of gene expression variation described here that elevate their potential importance above those of modules identified by unsupervised clustering methods. First, they are highly repeatable across studies in different locations and profiling different subsets of peripheral blood cells. This robustness is further indicated by the ability to capture the major axes of variation with as few as 10 BITs. Second, they are enriched for gene activities that are clearly related to biological functions, in particular inflammation, viral response, and signaling within B and T lymphocytes as well as neutrophils. The axes do not just reflect abundance of the cell types, but also the state of activity within them. Third, they are differentially expressed under a wide variety of blood disease conditions [12]. Other studies may highlight additional disease-related axes, and we note that our initial derivation of the first seven axes may have been biased by the diseases included in [12], but we have provided several lines of evidence that the axes described here are the major conserved ones in peripheral blood. The fact that healthy individuals vary with respect to axes of variation that are characteristic of disease suggests that the architecture of gene expression variation is primed to promote disease responses that are constrained along these axes.

An illustration of this principle is provided in Figure 4B, 4C which examines the relationship between gene expression and percent body fat in the form of a volcano plot [21] of significance against regression coefficient for all 7538 transcripts that are strongly correlated with the axes. Simply taking the 100 transcripts experiment-wide significant at negative log P greater than 4 does not show any marked enrichment for gene ontology categories. However, when transcripts in Axis 2 are highlighted, a clear and significant bias toward up-regulation of just this axis is observed. Not all genes associated with the axis show a significant change in expression, but the axis as a whole is modified. This implies that those individuals experiencing greater systemic immune stress due to their elevated body fat up-regulate a large fraction of transcripts associated specifically with these two axes. In the smaller Brisbane study, where no genes were significantly associated with BMI at experiment-wide levels, the Axis 2 score nevertheless is and the plots in Figure S7 clearly show the direction of effect.

We now discuss four key questions that arise from recognition of the potential for axes of covariance to shape individual responses to genetic and environmental challenges that tend to push them away from good health.

How generally are the axes associated with disease? Chaussabel et al identified 28 modules of gene expression that are contained within our axes, and proposed that different disease states may reflect differential activation of these modules [12]. In typical relatively small studies with tens rather than thousands of cases profiled, there will likely be a tendency to split common axes into subgroups, so more comprehensive analyses will be needed to establish to what extent autoimmune, infectious, and other blood states reflect specific activation of subsets of genes within the axes. Furthermore, it is possible that some disease responses involve differential expression that is not captured by the common axes of variation described here. At least 50 percent of the peripheral blood transcriptome is tightly correlated with these axes, and as much as two thirds is significantly correlated with them at false discovery rate levels, and hence disease responses will typically be influenced by activity of the regulators of the axes. This does not preclude other regulatory mechanisms from promoting disease orthogonally. A related question to this is whether similar axes of variation influence disease risk in other tissues.

To what extent does a person's healthy expression profile predispose them to blood diseases? It is typical when considering differential gene expression to assume that the most strongly up- or down-regulated genes are causally related to the disease. However, an alternative view is that they are part of a programmed protective molecular response, in which case it is the genes in the axis that are not induced that are more responsible for ill health. This view is encapsulated in the notion gaining traction [22], [23] that it is the variance of gene expression within specific modules or pathways that may be strongly associated with disease. Consequently, studies are needed that establish the statistical relationship between the Axes scores in health and disease. Are those who have a genetic predisposition to high scores along Axis 7 more at risk of viral infection, or more able to mount an efficient response? Are those who have naturally low Axes 5 scores at reduced risk of inflammatory disease, or particularly susceptible to stress-induced chronic conditions?

How heritable are the axes of variation? Family-based profiling of whole blood gene expression will answer this question. In CEPH lymphoblast cell lines, it was previously noted that the heritability of individual transcripts is highly significant for over half the transcriptome [24], [25]. We have estimated the twin-twin phenotypic correlation in the Brisbane twin study, and observe highly significant heritability both in whole blood (correlation coefficients from 0.34, Axis 2, to 0.61, Axis 3) and in LCL (in most cases more highly, likely reflecting reduced environmental variance in culture, an exception being Axis 3: Figure S8). Paradoxically, there is zero co-variance of the LCL and peripheral blood axis scores measured in the same individuals, consistent with the reported low level of genetic covariance between these tissues [18]. In other words, the axial nature of gene expression arises independently in blood and LCL, and yet is heritable in both cases. Further profiling of multiple fractionated leukocyte classes in a single cohort will be required to establish to what extent the whole blood profiles represent summation of the contributions of individual cell types and shared covariance between them. We also performed GWAS in the small Atlanta sample, but failed to identify any unambiguous regulatory loci, indicating that the regulation is complex, in line with that for other typical continuous traits. The corollary of heritability is plasticity, so it will also be important to establish to what extent Axes scores are maintained longitudinally in individuals, on the time scales of years and decades, but our analysis of the Snyderome [20](Figure 5), shows how the BIT might be used to report sub-clinical changes in individuals as they acquire infection or early onset of chronic diseases.

**Figure 6:**
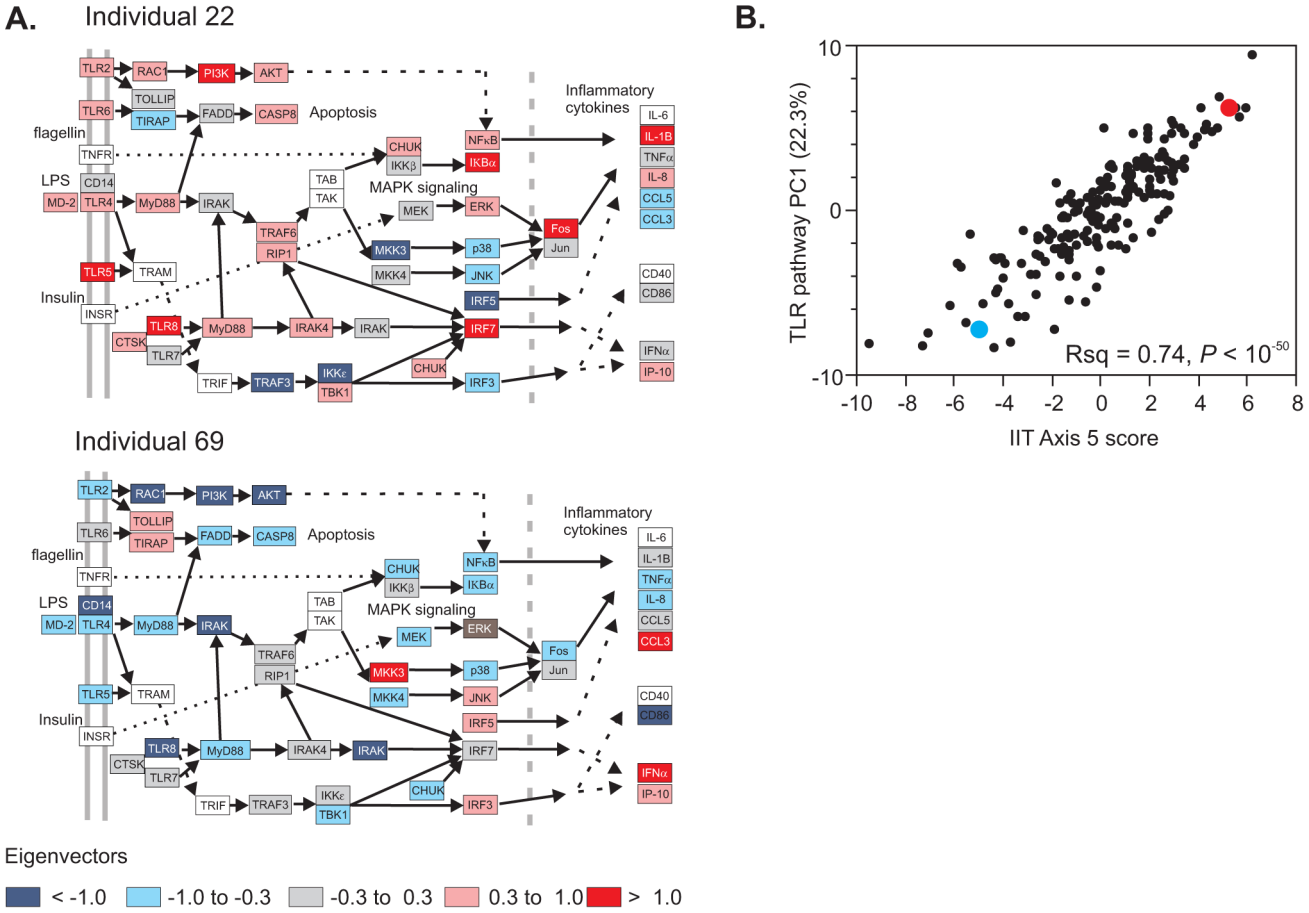
Pathway analysis of transcript abundance. The relative transcript abundance of 51 transcripts in the KEGG TLR signaling pathway (map04620) is shown for two representative divergent individuals (A). These genes are all co-regulated along Axis 5 (B), resulting in differential activity throughout the pathway. Two representative individuals at either extreme, indicated in red (individual 22) and blue (individual 69) in panel B, clearly differ with respect to which genes have high or low expression relative to regulation of apoptosis, MAPK signaling, and inflammatory cytokine production. This likely has consequences for the sensitivity of neutrophil and other immune cell function. Red, high expression; blue, low expression; gray intermediate, scaled as the eigenvector of PC1.

Finally, how does the existence of these major axes of variation constrain gene expression in biochemical pathways? It appears that the axis of variation of clearly defined pathways tend to be embedded within one of more of the Axes. For example, as shown in Figure 6, the Toll-Like Receptor signaling pathway is primarily explained by Axis 5. Individuals with high or low PC scores have average relative gene expression profiles indicated in panels 6A and 6C, which clearly shows an orchestrated patterning of covariance. This implies that a person's healthy status with respect to physiologically relevant pathways is likely to be a function of their genetic predisposition along the major Axes, as modified by the environment. This perspective frames the mathematical challenge to understand why evolution has resulted in co-regulation of the signal transduction apparatus, and to infer what the consequences are for biological responsiveness to perturbations that tend to promote or protect against disease.

## Methods

### Gene Expression Profiles

All datasets considered here were generated on Illumina HT12 v2 or v3 bead arrays, and unless otherwise indicated we used the raw gene expression measures reported in the published studies. For the Atlanta CHDWB study (described here for the first time), 189 individuals of mixed ethnicity and gender, between the ages of 26 and 75, donated 10 ml of blood during the course of their first visit to the Center for Health Discovery and Wellbeing. The whole blood samples were stored frozen in Tempus tubes, and RNA was extracted and hybridized at the Emory Biomarker Service Center under the direction of Dr Carlos Moreno. This dataset has been deposited in NCBI GEO under accession number GSE35846. The raw log base 2 expression profiles for 14,343 probes that are consistently expressed in the Morocco, Brisbane Red Cross, and CHDWB studies, were normalized by probe-level ANOVA fitting technical effects of date of hybridization and RNA quality, resulting in standardized normal transcript abundances, which were subsequently mean-centered across individuals. For direct comparison, the Morocco study values were also standard normalized and mean centered. Various other normalization procedures including analysis on the raw data, quantile normalization, and SNM [26] adjusting for additional technical and biological covariates including RNA quality, hybridization batch, cell counts, and serum chemistry, affect precise Axis scores, but do not significantly alter the scores derived from the BIT [27]. Age and Sex do not account for more than one or two percent of transcriptome variance and do not correlate with the axes described below. All individuals in Morocco and Atlanta are within the usual range of health for their populations, and include individuals with overt or sub-clinical metabolic disease but not any other known acute conditions.

The other studies considered here utilized whole blood in Tempus tubes (tuberculosis study [15]); whole blood in Paxgene tubes (Celiac disease study [16]; DILGOM Finnish study [17] and Brisbane twin study [18]); and leukocytes isolated in Leukolock filters (Morocco study [13] and Brisbane Red Cross study [14]). The original Chaussabel modularity study [12] was of Ficoll gradient sorted PBMC, profiled on Affymetrix U133A gene chips. Table S3 lists the relevant properties of each study, and raw data as well as anthropomorphic features mentioned in the text are available as Dataset S2 and from the authors' website at http://www.gibsongroup.biology.gatech.edu/supplementary-data.

### Derivation of the Axes

The primary objective of our analyses was to ask whether genes that are consistently co-regulated but that differ in expression between disease states, are also co-regulated in healthy people. If they are, it may imply that healthy people vary with respect to disease risk according to their normal profiles. The co-regulated modules were initially defined [12] using weighted gene co-expression analysis. We started by reasoning that co-regulation would be reflected in covariance defined by the major PC of gene expression of all of the genes in each module. We confirmed that this is the case in two distinct datasets from Morocco (lymphocytes) and Atlanta (whole blood), but also noted that these 28 PC1 scores themselves show a strong correlation structure within each dataset. Furthermore, the same modules cluster together in the two studies. Consequently, we collapsed the clustered modules into six meta-modules and redefined PC1. Hundreds of genes were highly correlated with each PC1 score in common in both studies. In each case, the top 10 genes were also very highly correlated (r>0.9) to one another, so we used these genes (which we call Blood Informative Transcripts, BIT) to define the common Axes of variation as described in detail below. Three more axes that are not observed in the Chaussabel modules were extracted from the residual unexplained variance. All of these BIT are very highly co-regulated in dozens of other blood gene expression studies that we have since examined, and some are also seen in cancer and other cell lines. Multivariate regression shows that 5 of the axes are associated with gene expression transcriptome-wide as shown in Figure S3B. The next five paragraphs describe details of the analytical pipeline.

For each of the CHDWB and Morocco studies, we extracted all of the probes associated with each of the genes listed in the 28 Modules defined in [12], and generated Principal Component 1 (PC1) for each module. Table S4 lists the amount of variance explained by each PC1 (27.7% and 25.0% in Atlanta and Morocco respectively), which in each case is considerably greater than observed with random samples of a similar number of probes (15.8%±2%). Figure 2A and the bottom right panel in Figure S3C plot the loadings for the first two PC for two typical modules, numbers 2.6 and 3.5, showing that most genes are positively co-regulated (red lines extend to the right). Figure 1A shows that the correlation structure of these scores is highly conserved in the sense that the same modules cluster by similarity, allowing us to define Meta-Modules by amalgamation of Modules 1.7, 2.1, 2.4, 2.8 and 3.8 (Axis 1), 1.2, 2.2, 2.3 and 2.5 (Axis 2), 1.1 and 1.3 (Axis 3), 1.4, 2.7, 2.9, 2.11, 3.4, 3.6 and 3.9 (Axis 4), 1.5, 2.6, 3.2, 3.3, and 3.5 (Axis 5), whereas Module 3.1 defines both Axes 6 and 7. Modules 1.6, 1.8, 2.10 and 3.7 also show co-regulation in healthy people, but are correlated either with multiple Meta-Modules, or split into two-or-more sub-modules, so these were not included in further analyses. Axis 6 appeared serendipitously, as we noticed when generating the module PCs that a small number of individuals were consistent outliers along the second PC for several of them, notably 2.7, 2.11 and 3.8 in Morocco and to a lesser extent CHDWB. A search for genes that are differentially expressed in these individuals uncovered a novel set of highly co-regulated transcripts, the covariance of which was then used to define Axis 6, which turns out to be as highly conserved as the other major axes.

Next, we generated PC1 for all genes in each Meta-Module and evaluated their relationship to the first 5 Principal Components (Prin1–Prin5) of the full dataset of 14,343 transcripts. Table S5 shows that overall Prin1 is strongly positively correlated with Meta-Module 4 PC1 in both datasets, and also negatively correlated with Meta-Module 2. Overall Prin2 is positively correlated with Meta-Module 5, and negatively correlated with Meta-Module 1. There is little consistency to the remainder of the correlation matrix of Meta-Module PCs and whole transcriptome PCs. Interestingly, Axis 6 is strongly negatively correlated with the 3^rd^ and 4^th^ PC in the Atlanta CHDWB and Moroccan studies respectively. It is thus surprising that Axis 6 does not correspond to any of the modules described by Chaussabel et al. [12].

We then performed a multiple regression using the ANOVA routine in JMP Genomics, generating the list of probes and genes that are highly significantly correlated with each axis (at single axis Bonferroni significance, p<3.5×10^−6^) while adjusting for the other axes in the model. All probes that were significant in both the CHDWB and Morocco datasets are listed in Dataset S1 – these define the Axes. The linear model was of the form Y_jk_ = μ+Σa_i_(PC1_i_)+ε where the expression Y of the *j*th gene in the *k*th individual is a function of the sum over all *i* = 1 to 7 Axis PC1 scores, and the error ε is assumed to be normally distributed with mean of zero. (We also generated axes based on the univariate correlations, and these agree for the most part with the axes defined here, but as expected do not separate as cleanly). Figure S3B shows *a*_i_ from the two studies plotted against one another, demonstrating that the association of each transcript with each axis is remarkably highly conserved between the two studies, in the case of Axes 1, 3, 4, 5 and 7 transcriptome-wide (whereas Axes 2 and 6 associated genes form more distinct clusters).Table S4 lists the amount of variance explained by PC1 for the first 175 probes on each list of the Axis-defining genes (the most significant ones) in Dataset S1 (or all 118 probes for Axis 3). This number of probes was chosen as the average number representing each of the Chaussabel modules, so facilitates comparison with the module scores. In every case the Axis-associated genes are more tightly co-regulated than the original modules: on average, 57.2% and 50.2% of the variance in Atlanta and Morocco is captured by the 175-probe Axis PC1, twice as much as for modules of the same average size. All significant *a*_i_ from the two studies were cross-matched to generate the probe lists that define each Axis, with the additional constraint that only positive associations are included (these are the majority in all cases). Figure S3C confirms that that in all cases the genes are positively co-regulated and that PC1 explains much more of the variance than any other component.

In order to generate the BIT lists, we ordered the shared Axis-defining genes by the sum of the significance negative log p-values from the multiple linear regression described above, for the two studies, and picked the top 10 probes that were not also highly significantly associated with any other axes. These probes are listed in Table S1. In every case, they explain 50% or more of the variance of the BIT, whereas 100 random permutations of 10 probes never explained more than 35% (Figure S3A for CHDWB; Figure 2E for Morocco). Axis PC1 scores were also generated using just the top 5 probes, or an expanded set of 20 probes (Dataset S4), and in all cases the correlation between the 10-BIT and either 5-BIT or 20-BIT score was greater than 0.95, and in 31 of 36 cases it was greater than 0.98, three of the exceptions involving Axes 8 and 9, and the other two the 5-BIT comparison. Correlation coefficients between 10-BIT scores and PC1 scores based on the 250 most strongly associated probes (or all 118 for Axis 3 and 221 for Axis 7) were similarly all greater than 0.88, p<10^−70^, with median of 0.96. We chose 10 BIT since this number is convenient for generation of a target qRT-PCR panel that captures all Axes [28]. Each of the axis scores in all 7 studies considered here are, unsurprisingly, very similar to the axis scores based on a separate list of BITs derived from the highest univariate correlations. It is likely that further analyses of more datasets may identify even more diagnostic probes, but note that most of the probes in each BIT set already have correlation coefficients greater than 0.9 for that set.

We then asked whether there are additional shared major covariance modules unrelated to Axes 1 through 7, by fitting the PC1 scores for these Axes and performing PCA on the residuals, for both the CHDWB and Morocco studies separately. Residual PCs 2 and 3 had highly correlated loadings across all probes comparing the two studies (though the PC rank numbers were switched), so the above process was repeated giving rise to Axes 8 and 9. That is, we performed multiple regression on the original datasets with the first 7 axis scores plus residual PC2 and PC3, then cross-matched probes that were Bonferroni significant with residual PC2 in CHDWB and residual PC3 in Morocco to obtain the Axis 8 genes, and with residual PC3 in CHDWB and residual PC2 in Morocco to obtain the Axis 9 genes. CHDWB residual PC1 loadings were mildly correlated with Morocco residual PC4, but very few genes were significantly cross-matched in a similar analysis and this potential axis was ignored. There may be additional axes but since Axis 8 and Axis 9 only explain an extra few percent of the total variation, these nine axes appear to be the major conserved axes in our datasets. Axes 8 and 9 are noticeably weaker (explain less of the covariance of associated transcripts) so are not presented in all figures and analyses.

### Statistical Analyses

All statistical manipulations, as well as correlation analyses leading to the findings reported in the figures were performed in JMP Genomics v5.0 (SAS Institute, Cary, NC). See Figure or Supporting Information Legends for details of individual analyses, and Dataset S2 for data required to repeat the linear regression analyses of trait associations. Panels in Figure 2 report ANOVA contrasting the effects of location in Morocco, or TB status, on Axis scores (PC1 for the 10 BIT) for each axis separately. The ToppFun analysis [19] was performed initially in February 2012, and again on November 27, 2012, using the online resource at http://toppgene.cchmc.org/enrichment.jsp, with similar results. Only the top 500 probes were included for each analysis, so as to ensure comparability across Axes with different numbers of genes, but modification of the number of genes included did not meaningfully affect the core results. Since some probes are duplicated while others are not annotated to genes, the numbers listed are less than 500. ToppFun computes gene set enrichment relative to the full set of annotated genes in the genome, and since the database is updated regularly specific results change subtly over time. Detailed results can be viewed as Dataset S3, which clarifies the strength of evidence (number of genes in the Axis and genome, p-values) derived from enrichment for GO terms and abnormal mouse phenotypes. These generally agree, but also provide slightly different perspectives. The Transcription Factor Binding Site and miRNA binding site predictions are purely computational, and only show enrichment for a subset of genes in each Axis. The human disease associations are only with a few genes in each case. None of these analyses should be interpreted too literally, they are provided simply to illustrate the non-random nature of the Axes.

For the Synderome analysis [20], we downloaded the RNA-Seq BAM files from GSE33029 and obtained the FPKM for all 25,227 transcripts reported. 6,862 low expression genes were deleted, and the remaining 18,365 measures were log2 transformed, the profiles were median centered, each probe was standardized to z-scores, and 83 of the 90 BIT genes present in the profiles were extracted. As with all other datasets, the first PC for each BIT-defined axis was determined from these available transcript abundance measures, and they are plotted across the time course in Figure 4. One sample, GSN818565 (Day 21) was excluded due to very low counts.

### Ethics Statement

All samples reported for the first time in this study were obtained under written informed consent for participation in the Center for Health Discovery and Well Being study with the approval of the Institutional Review Boards of Emory University and the Georgia Tech.

## Supporting Information

Dataset S1 List of genes associated with each of the 9 axes in both Atlanta and Morocco. The 9 sets of columns show the Illumina Probe identifier, the Gene name, and the negative logarithm of the p-value (NLP) for the association of the transcript with the axis score (PC1) in the multiple regression with all 9 axes in Atlanta (NLP_ATL) and Morocco (NLP_MOR). Only probes significant at the Bonferroni adjusted significance level (NLP>5.53) in both studies are included, and they are sorted by average NLP.
(XLSX)

Dataset S2 List of covariates and Axis scores for five studies. Each sheet shows the individual identifier and coariates including Age, BMI, Percent Body Fat, Gender, Ethnicity, Location, and 8 types of cell count for the CHDWB; Location, Gender, Ethnicity and Age for Morocco (13); Gender, Age and BMI for Brisbane Red Cross (14); Study ID and PC1 for the TB status signature reported in the Berry et al study [15]; and the twin identifier for the Brisbane Twin study [18]. Axis scores are listed as PC1 for the 10 Blood Informative Transcripts (BIT).
(XLSX)

Dataset S3 ToppFun gene ontology analysis of each of the 9 Axes. The top 500 genes listed in Dataset S1 were entered into ToppFun [19] on November 27, 2012. This sheet reports the major independent enrichments scores for each axis with respect to Gene Ontology classes (GO), Human or Mouse Phenotypes (HP or MP), Transcription Factor or miRNA binding sites, and human diseases where present. P-values in column C are Bonferroni-adjusted, and numbers in column B show the number of genes in the Axis/Number of genes in the genome annotated to the relevant term. Total number of genes per axis is less than 500 because not all probes are annotated and some are duplicated, and for Axes 2, 3, 7 and 9 fewer than 500 are associated with the Axis at Bonferroni significance level in both studies.
(XLSX)

Dataset S4 Axis scores based on 5, 10, 20 or up to 250 BIT. The two spreadsheets show the PC1 scores based on the 5, 10, 20 or up to 250 most strongly associated probes listed in Dataset S1, for each axis. Within an axis, all four scores are highly correlated.
(XLSX)

Figure S1 (A) Scatterplot of Eigenvalues for each of 14,343 transcripts on PC1 in the CHDWB and Morocco studies. While there is a strong correlation between the loadings for 90% of the transcripts, 10% all have higher values in the CHDWB study. This PC is highly correlated with Axis 4. (B) Lack of orthogonality of PC across studies. Each of PC2 through 5 in the CHDWB study is as strongly correlated with two PC in the Morocco study (for example, CHDWB PC3 with Morocco 2 and 4) reducing the utility of study-specific components of variation for comparative purposes.
(PDF)

Figure S2 Independent evidence that the first 7 Axes are the major axes of covariance. For any set of covarying transcripts, some individuals will be expected to have low values of expression for multiple transcripts in the set, and these will be enriched in the low-expression transcripts of that individual. We thus reasoned that clustering of the variance components of low-expression genes should independently identify the major axes of covariance. For each individual in the Morocco study, up to 100 transcripts that are outliers for low expression (that is, more than two standard deviations below the mean standardized expression values of all transcripts in the individual sample) were identified. PC1 was computed for these transcripts, two-way hierarchical clustering of the 189 scores (rows) across the 189 individuals (columns) is shown in the upper plot (A). Nine distinct clusters are observed, seven of which uniquely cluster with the BIT score for one of the first seven Axes (indicated by arrowheads). The rows marked Axis 2b appear to be a subset of Axis 2, and the small unmarked cluster of rows between Axes 3 and 5 may define a new Axis, but further analysis indicates that the scores are driven by very low expression in just the indicated individuals. The lower panel (B) shows the heat map generated by only including the two individuals whose low-expression PC1 most strongly correlates with the respective BIT. Only one individual is close to BIT 7 (which was not uniquely represented by one of the Chaussabel modules), and no individuals are close to BIT 8 or BIT 9. These results are consistent with the first 7 Axes being the major axes of variation, though not necessarily the only ones.
(PDF)

Figure S3 Blood Informative Axis scores. (A) Each plot shows the PC1 loadings of the 10 Bit transcripts in the CHDWB study on the right, and the individual PC1 and PC2 scores on the left. The same result for a typical random set of 10 probes is included as well. Panel J shows a histogram of the percent variance explained by PC1 for 100 random sets of 10 transcripts, relative to that observed for each of the BIT Axes, which are unambiguous outliers. (B) Pairwise comparison of the multiple regression coefficient for each transcript in the CHDWB/Atlanta and Morocco studies for each Axis, showing transcriptome-wide similarity for Axes 1, 3, 4, 5 and 7, whereas a subset of transcripts are clearly more strongly associated with Axes 2 and 6. (C) Principal component analysis of the 175 probes most strongly associated with each Axis, compared with a similar plot for a typical Chaussabel module, 3.5. In each case, the histogram of eigenvalues to the left shows how PC1 (the Axis score) dominates the covariance. The amount of variation explained by Axes and Modules in each study is compared in Table S4.
(PDF)

Figure S4 The covariance of BIT Axes is somewhat study-specific. Panels A and D show the correlation between the BIT Axis scores (namely, PC1 for the 10 probes as shown in Figure S2) for all individuals in CHDWB and Morocco respectively. Panels B and C (or E and F) then show a typical result of splitting each study into two halves, recomputing the Axis scores, and clustering the correlations between them. Visually, the split studies resemble the whole study in each case, and this is also true of the DILGOM study. Panel G shows that the BIT Axis scores themselves are very highly correlated for individuals in one half compared with their values in the whole study but uncorrelated with each of the other Axes. Panel H shows the pairwise correlations of Axis scores for each study and two halves, with the significance of the study differences (ANOVA on the effect of the three studies, namely with 2 degrees of freedom for study and 6 for error). * p<0.05; * p<0.01; ** p<0.001; *** p<0.0001; **** p<0.00001. Axes 8 and 9 were excluded from this analysis for clarity, since they are less robust than the first 7 Axes. Other partitions of each dataset give similar results.
(PDF)

Figure S5 Similarity of correlation structure among Axis scores (PC1 for each of the 10 BIT for each axis) across 6 independent whole blood datasets. In each of the Atlanta CHDWB, Morocco, Brisbane Red Cross (our studies), Celiac Disease, Tuberculosis, and Finland (DILGOM) (performed by others), Axes 1 and 3, and Axes 5 and 7, are to some extent positively correlated. Negative correlations arise in some instances (Axes 1 and 5, and Axes 2 and 4). The reason for the generally more positive correlations in the Celiac study is unclear, but it is noteworthy that all BIT tend to show stronger internal correlations in that study as well (Table S2).
(PDF)

Figure S6 Cell counts correlate with specific Axes, but do not explain the axes. (A) T-lymphocyte count is positively correlated with Axis 1, but in part due to high scores of individuals with large T-cell counts. (B) Neutrophil counts are correlated with Axis 5, explaining 34% of the variance. Red females, blue males. (C) However, removing the effect of cell counts during normalization with the SNM algorithm (23) has very little effect on axis definition, as the Axis scores match one-to-one with those derived without such normalization [29].
(PDF)

Figure S7 Replication of the association of BMI with Axis 2 in the Brisbane Red Cross study. Panels A and B show that BIT 2 correlates with both BMI and %BF in Atlanta CHDWB (%BF differs significantly between men and women). The same association is observed in Brisbane (C), where %BF data was not gathered. The volcano plots of significance (NLP, negative log_10_ of the p-value) against estimated BMI effect (the slope of the regression of BMI on Axis score) is also show how Axis 2 (D), but not Axes 1 or 6 (E,F), is strongly skewed to up-regulation of genes in Axis 2 in higher BMI individuals. The smoother shape of the curves relative to those in the CHDWB in Figure 3 is due to the lesser influence of technical and geographic factors on gene expression and reduced the estimated BMI effect for most transcripts.
(PDF)

Figure S8 Heritability and differentiation of LCL and Peripheral Blood (PB) in the Brisbane twin study (18). PC1 scores for the 10 BIT per axis were computed for each dataset and are provided in Dataset S2. The percent variation of these BIT explained by PC1 is indicated in panel A, along with the significance (P-value) of the correlation between individual scores for LCL and PB. Six of the first seven BIT are also observed in lymphoblast cell lines (LCL), the exception being Axis 2. Nevertheless, there is no correlation between the axis scores for LCL and PB, as indicated in the heat map in panel B. Also, each of Axes 1,3,4,5 and 7 are highly correlated in LCL suggesting that they represent a single shared covariance structure. These results suggest that the whole blood profiles may arise by summation of contributions of different blood cell types. Although the LCL axes are different from PB ones, they also show significant heritability since the twin-twin phenotypic correlations are high for five of the axes as indicated in panel C. These were computed on the PC1 scores for the respective Axes, after removal of 2 outliers (and their twin partners) who are more than two standard deviations from the mean for multiple axes, indicated in italics on Dataset S2. The strongest twin-twin correlation is shown in panel D. These results imply that genetic factors contribute to the establishment of the shared covariance in individual cell types, and in peripheral blood as a whole.
(PDF)

Table S1 List of Blood Informative Transcripts including the Probe ID for the Illumina Human_HT12 bead chips.
(DOCX)

Table S2 Percent variance explained by PC1 for the 10 Blood Informative Transcripts for each Axis, in each of the 7 studies, showing high replication of their co-regulation.
(DOCX)

Table S3 List of 9 studies referred to in the paper, showing the Location of the population, number of samples, source of blood RNA, GEO or ArrayExpress accession number, and reference in this paper (CHDWB is reported for the first time).
(DOCX)

Table S4 Percent variance explained by PC1 for the entire set of probes annotated to the Chaussabel module genes (PVE_CHD and PVE_MOR refer to CHDWB Atlanta, and Morocco studies respectively), also showing the number of probes and genes, the Axis each module associates with, and the average for each measure. The bottom rows show the same values for the 175 most strongly associated axis genes for the first 7 axes from Dataset S1, and their averages.
(DOCX)

Table S5 Correlation of Overall transcriptome PCA with Meta-Modules PC1 in Atlanta and Morocco. The values are the correlation coefficients between the first Principal Component for each of the indicated Meta-Modules and for Axis 6, and the first 5 Principal Components (Prin1-5) of the entire gene expression dataset for both the CHDWB Atlanta, and Morocco, studies separately. Bold values are consistent between the two studies, noting that Prin3 and Prin4 negatively correlate with Axis 6 respectively.
(DOCX)
