# Supplementary figures and images for: Blood-Informative Transcripts Define Nine Common Axes of Peripheral Blood Gene Expression
Source: PLoS Genet. 2013 Mar 14;9(3):e1003362. doi: 10.1371/journal.pgen.1003362 (PMC3597511; doi:10.1371/journal.pgen.1003362)

Supplementary Figure S1 Preininger et al, 2012

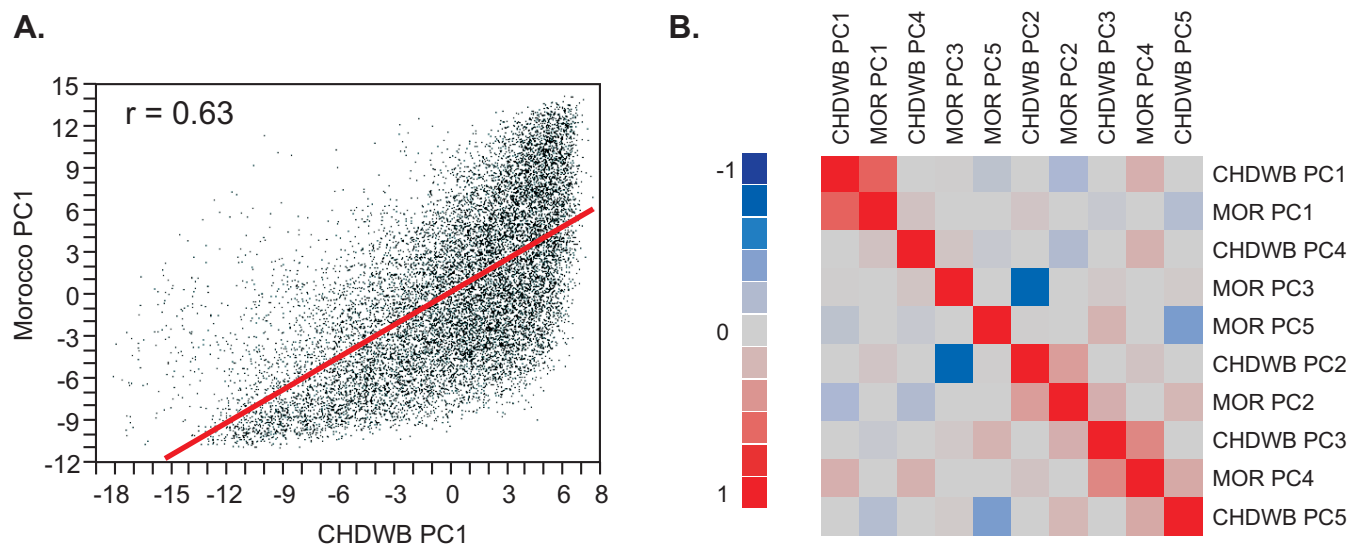

Supplement: Figure S1 — (A) Scatterplot of Eigenvalues for each of 14,343 transcripts on PC1 in the CHDWB and Morocco studies. While there is a strong correlation between the loadings for 90% of the transcripts, 10% all have higher values in the CHDWB study. This PC is highly correlated with Axis 4. (B) Lack of orthogonality of PC across studies. Each of PC2 through 5 in the CHDWB study is as strongly correlated with two PC in the Morocco study (for example, CHDWB PC3 with Morocco 2 and 4) reducing the utility of study-specific components of variation for comparative purposes. (PDF) [file pgen.1003362.s005.pdf]

Supplementary Figure S2. Preininger et al, 2012

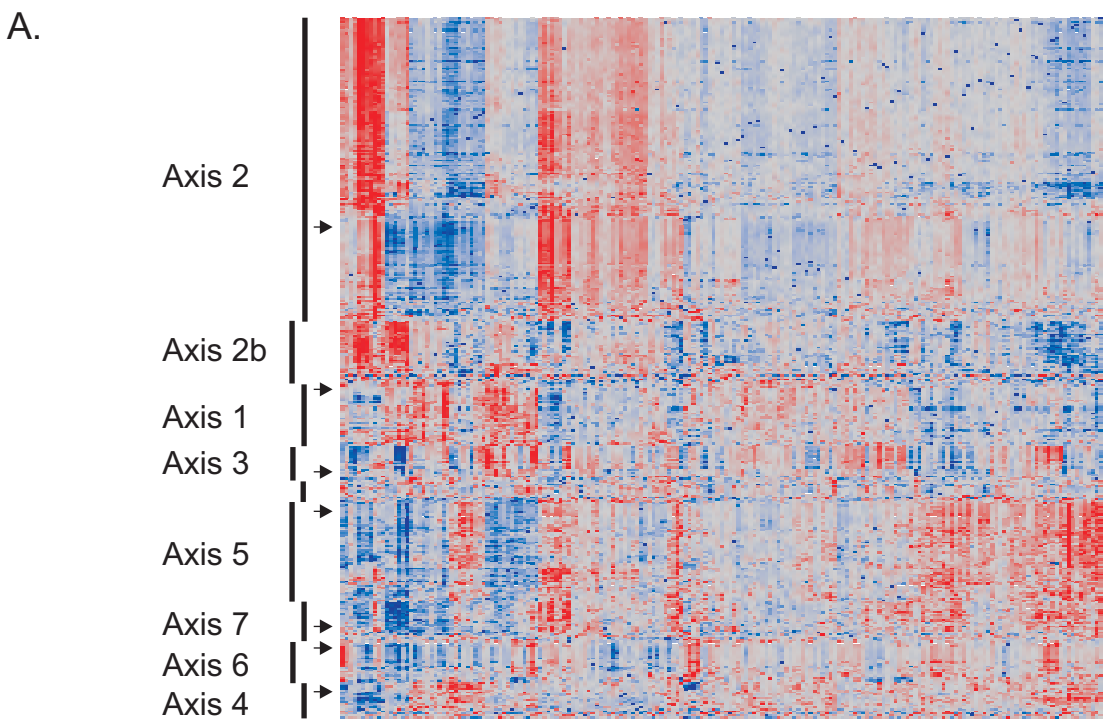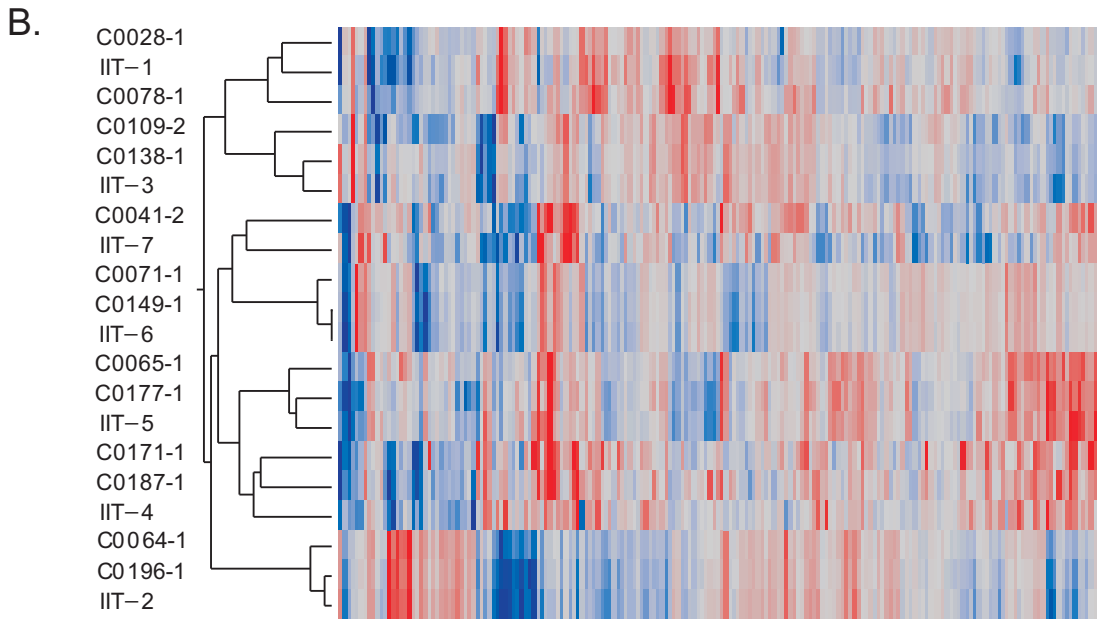

Supplement: Figure S2 — Independent evidence that the first 7 Axes are the major axes of covariance. For any set of covarying transcripts, some individuals will be expected to have low values of expression for multiple transcripts in the set, and these will be enriched in the low-expression transcripts of that individual. We thus reasoned that clustering of the variance components of low-expression genes should independently identify the major axes of covariance. For each individual in the Morocco study, up to 100 transcripts that are outliers for low expression (that is, more than two standard deviations below the mean standardized expression values of all transcripts in the individual sample) were identified. PC1 was computed for these transcripts, two-way hierarchical clustering of the 189 scores (rows) across the 189 individuals (columns) is shown in the upper plot (A). Nine distinct clusters are observed, seven of which uniquely cluster with the BIT score for one of the first seven Axes (indicated by arrowheads). The rows marked Axis 2b appear to be a subset of Axis 2, and the small unmarked cluster of rows between Axes 3 and 5 may define a new Axis, but further analysis indicates that the scores are driven by very low expression in just the indicated individuals. The lower panel (B) shows the heat map generated by only including the two individuals whose low-expression PC1 most strongly correlates with the respective BIT. Only one individual is close to BIT 7 (which was not uniquely represented by one of the Chaussabel modules), and no individuals are close to BIT 8 or BIT 9. These results are consistent with the first 7 Axes being the major axes of variation, though not necessarily the only ones. (PDF) [file pgen.1003362.s006.pdf]

# Supplementary Figure S4 Preininger et al, 2012

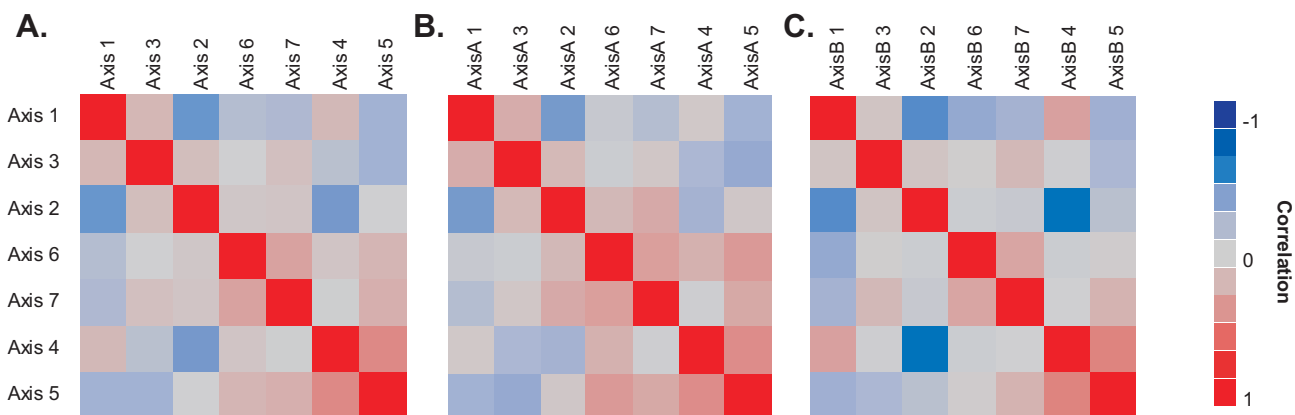

Atlanta CHDWB

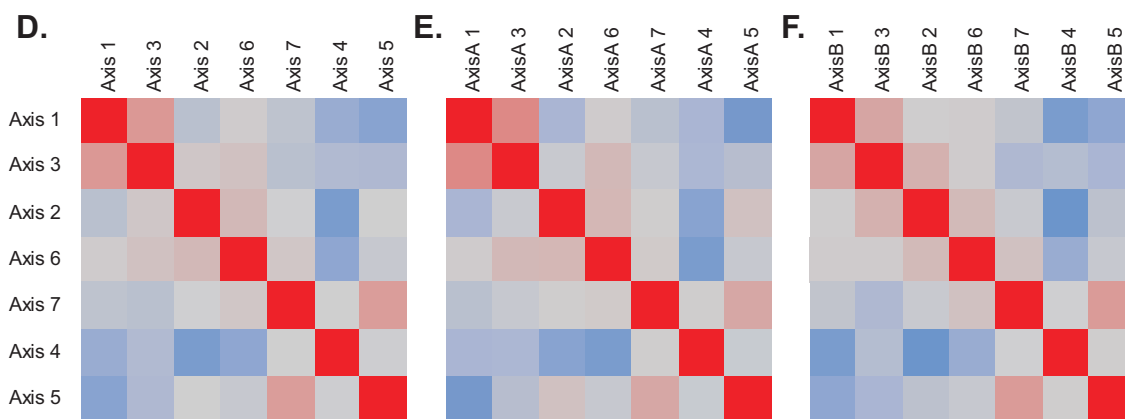

Morocco

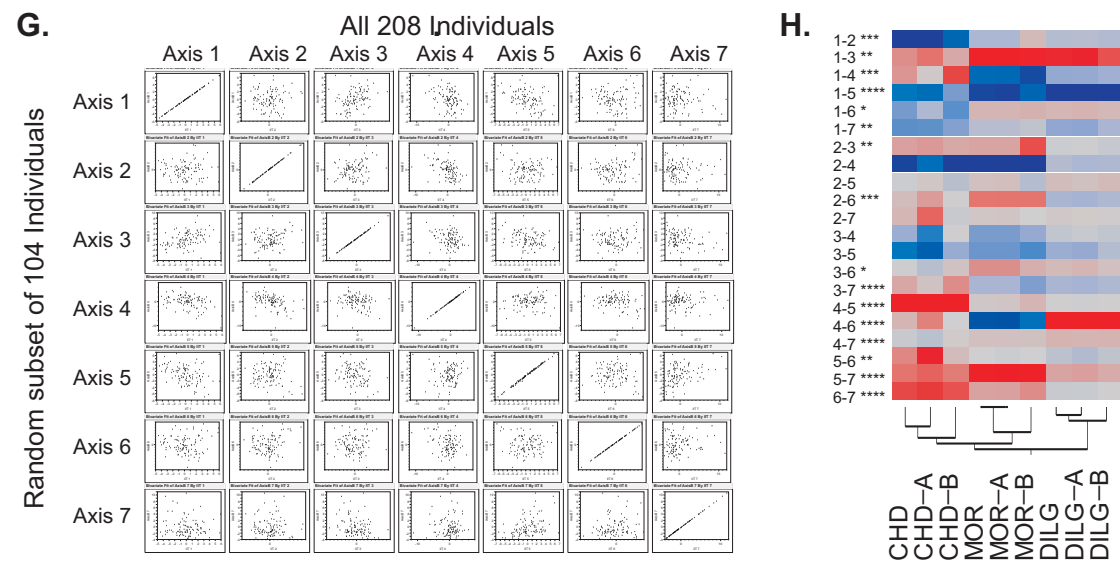

Supplement: Figure S4 — The covariance of BIT Axes is somewhat study-specific. Panels A and D show the correlation between the BIT Axis scores (namely, PC1 for the 10 probes as shown in Figure S2) for all individuals in CHDWB and Morocco respectively. Panels B and C (or E and F) then show a typical result of splitting each study into two halves, recomputing the Axis scores, and clustering the correlations between them. Visually, the split studies resemble the whole study in each case, and this is also true of the DILGOM study. Panel G shows that the BIT Axis scores themselves are very highly correlated for individuals in one half compared with their values in the whole study but uncorrelated with each of the other Axes. Panel H shows the pairwise correlations of Axis scores for each study and two halves, with the significance of the study differences (ANOVA on the effect of the three studies, namely with 2 degrees of freedom for study and 6 for error). * p<0.05; * p<0.01; ** p<0.001; *** p<0.0001; **** p<0.00001. Axes 8 and 9 were excluded from this analysis for clarity, since they are less robust than the first 7 Axes. Other partitions of each dataset give similar results. (PDF) [file pgen.1003362.s008.pdf]

Supplementary Figure S5 Preininger et al, 2012

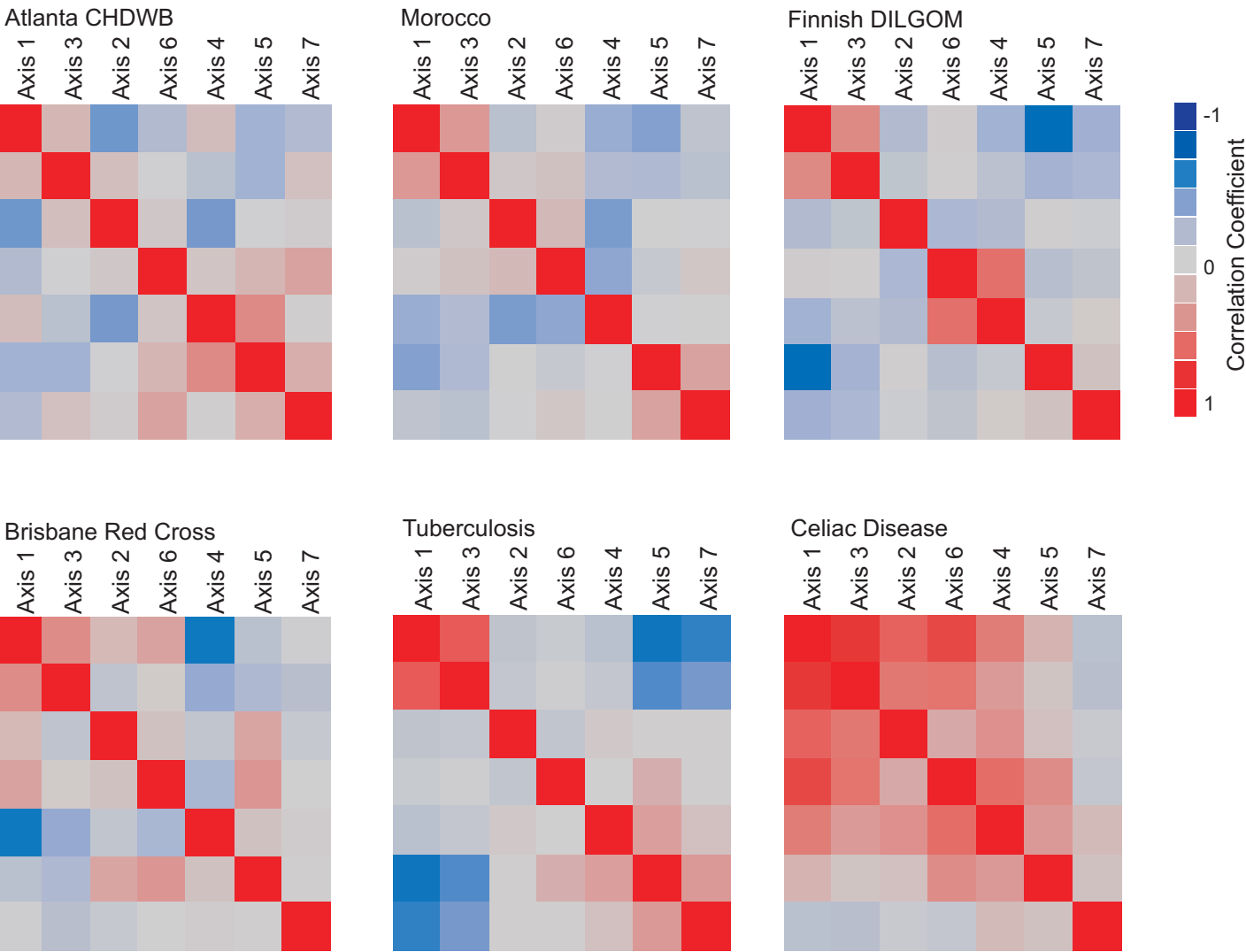

Supplement: Figure S5 — Similarity of correlation structure among Axis scores (PC1 for each of the 10 BIT for each axis) across 6 independent whole blood datasets. In each of the Atlanta CHDWB, Morocco, Brisbane Red Cross (our studies), Celiac Disease, Tuberculosis, and Finland (DILGOM) (performed by others), Axes 1 and 3, and Axes 5 and 7, are to some extent positively correlated. Negative correlations arise in some instances (Axes 1 and 5, and Axes 2 and 4). The reason for the generally more positive correlations in the Celiac study is unclear, but it is noteworthy that all BIT tend to show stronger internal correlations in that study as well (Table S2). (PDF) [file pgen.1003362.s009.pdf]

Supplementary Figure S6    Preininger et al, 2012

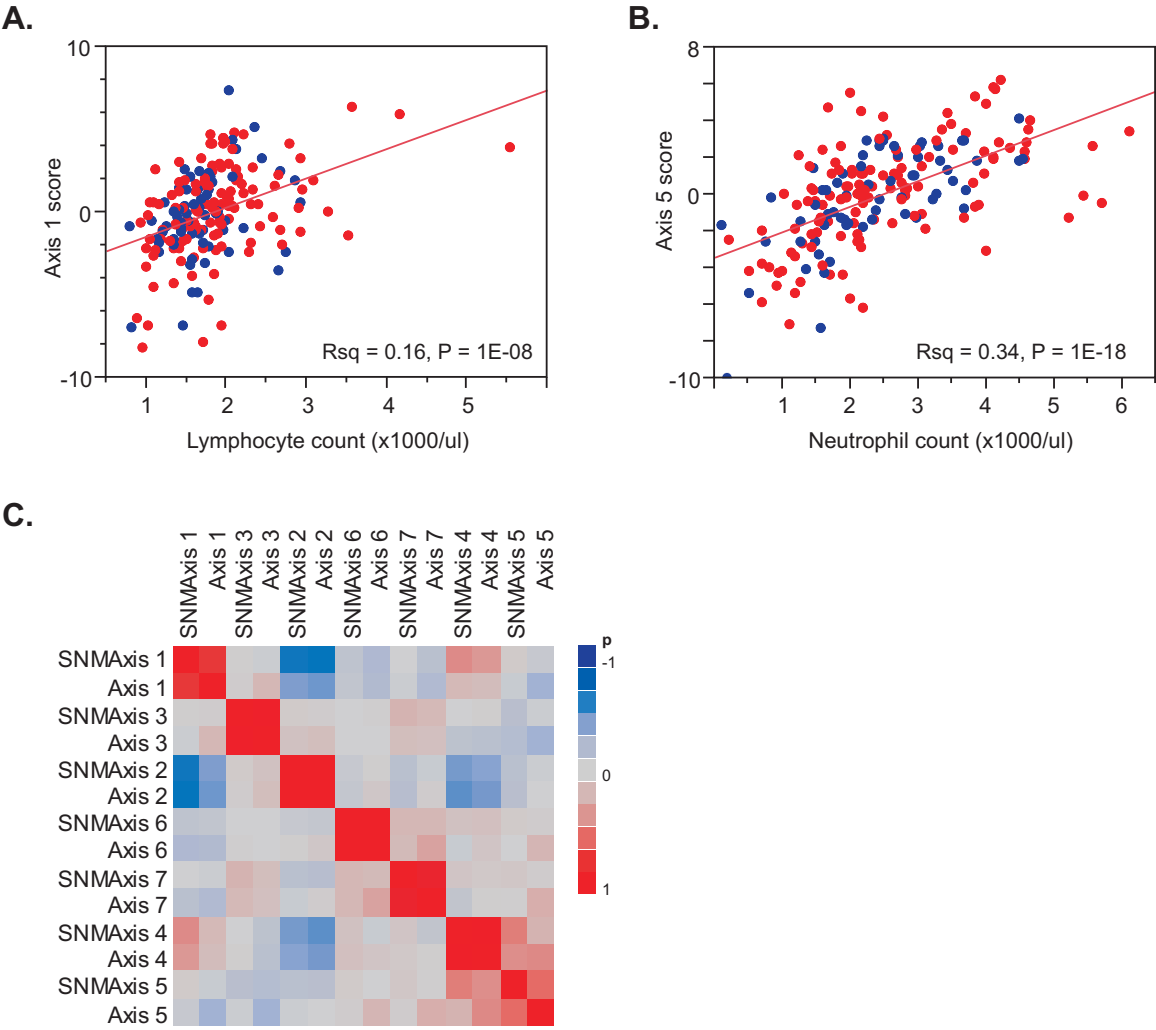

Supplement: Figure S6 — Cell counts correlate with specific Axes, but do not explain the axes. (A) T-lymphocyte count is positively correlated with Axis 1, but in part due to high scores of individuals with large T-cell counts. (B) Neutrophil counts are correlated with Axis 5, explaining 34% of the variance. Red females, blue males. (C) However, removing the effect of cell counts during normalization with the SNM algorithm (23) has very little effect on axis definition, as the Axis scores match one-to-one with those derived without such normalization [29]. (PDF) [file pgen.1003362.s010.pdf]

Supplementary Figure S7 Preininger et al, 2012

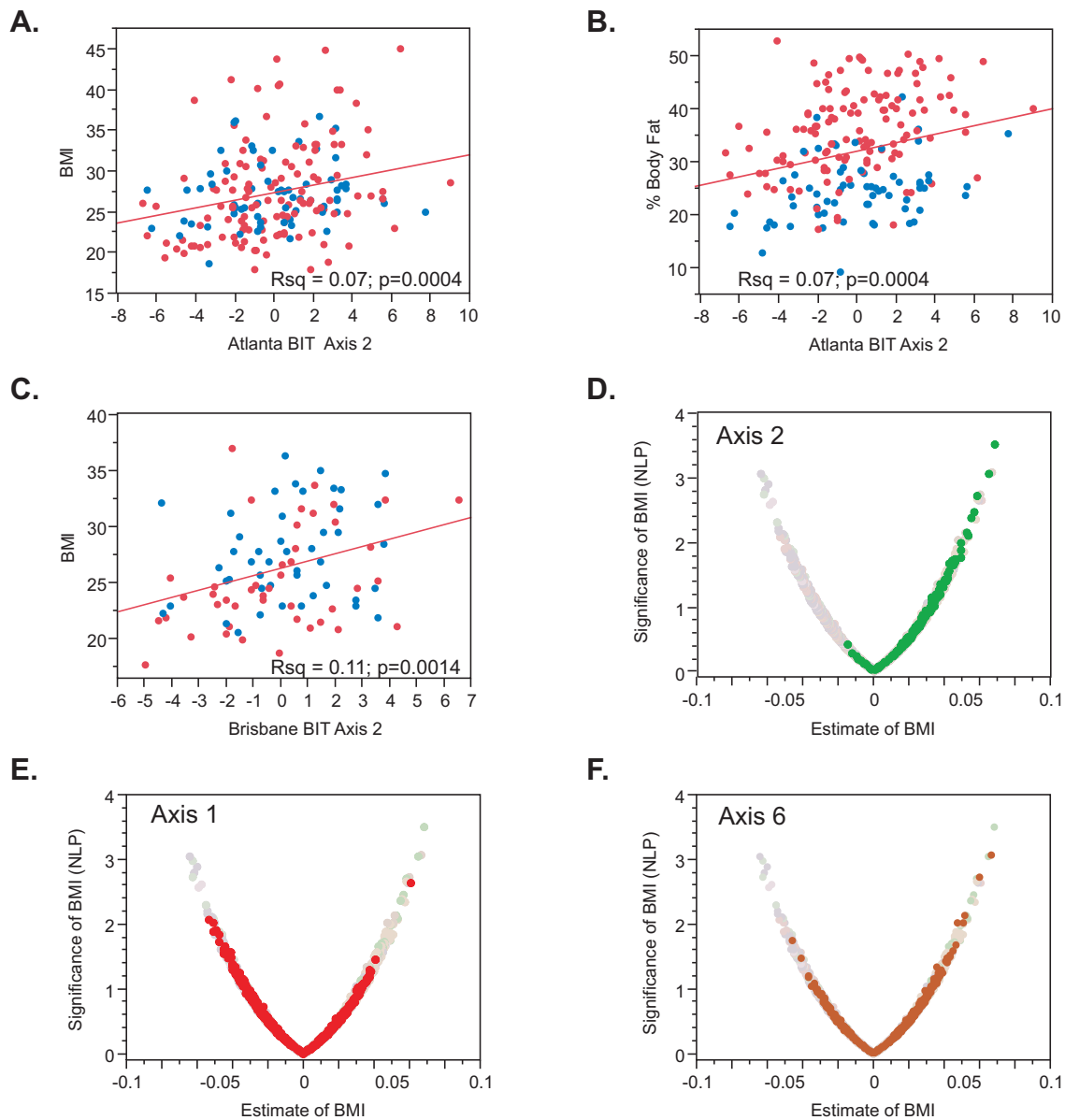

Supplement: Figure S7 — Replication of the association of BMI with Axis 2 in the Brisbane Red Cross study. Panels A and B show that BIT 2 correlates with both BMI and %BF in Atlanta CHDWB (%BF differs significantly between men and women). The same association is observed in Brisbane (C), where %BF data was not gathered. The volcano plots of significance (NLP, negative log10 of the p-value) against estimated BMI effect (the slope of the regression of BMI on Axis score) is also show how Axis 2 (D), but not Axes 1 or 6 (E,F), is strongly skewed to up-regulation of genes in Axis 2 in higher BMI individuals. The smoother shape of the curves relative to those in the CHDWB in Figure 3 is due to the lesser influence of technical and geographic factors on gene expression and reduced the estimated BMI effect for most transcripts. (PDF) [file pgen.1003362.s011.pdf]
