# Supplementary material for: Blood-Informative Transcripts Define Nine Common Axes of Peripheral Blood Gene Expression
Source: PLoS Genet. 2013 Mar 14;9(3):e1003362. doi: 10.1371/journal.pgen.1003362 (PMC3597511; doi:10.1371/journal.pgen.1003362)

Supplementary Figure S3A Preininger et al, 2012

BIT Axis 1

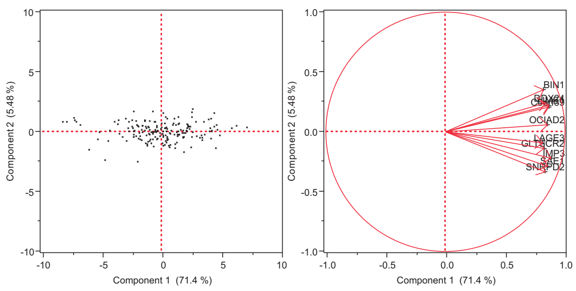

BIT Axis 2

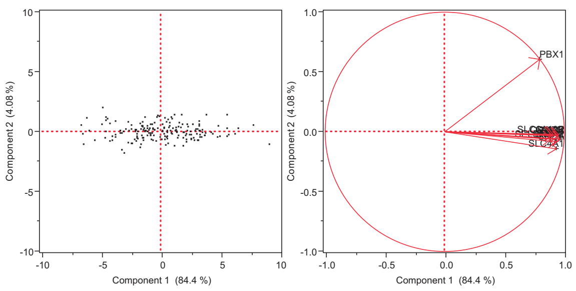

BIT Axis 3

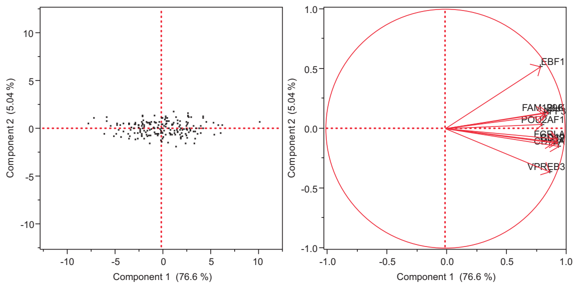

BIT Axis 4

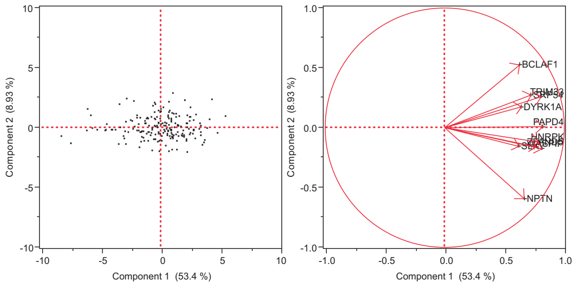

BIT Axis 5

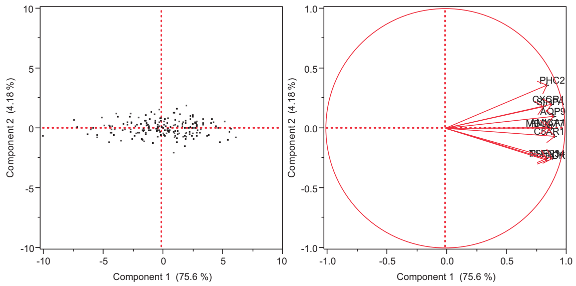

BIT Axis 6

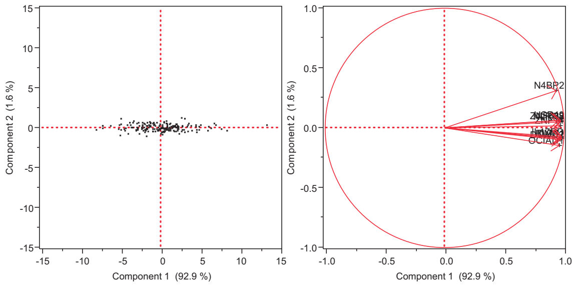

BIT Axis 7

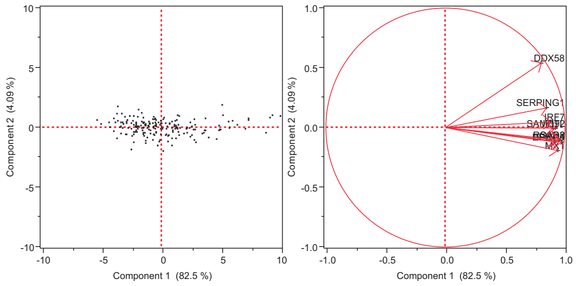

BIT Axis 9

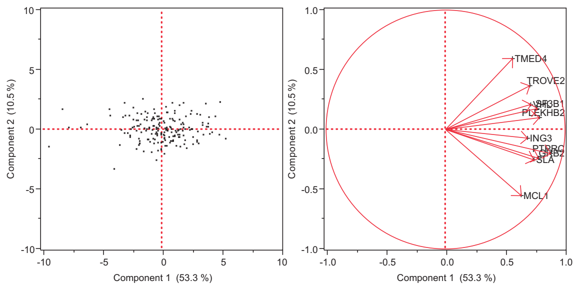

Typical Random Set

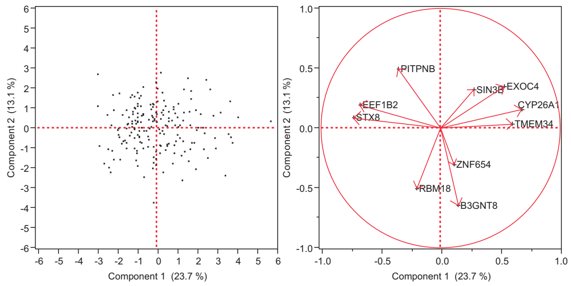

J. Variance explained by BITs

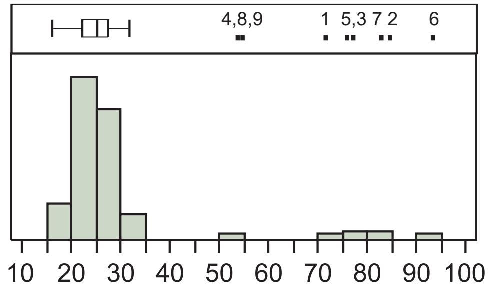

Supplementary Figure S3B   Preininger et al, 2012

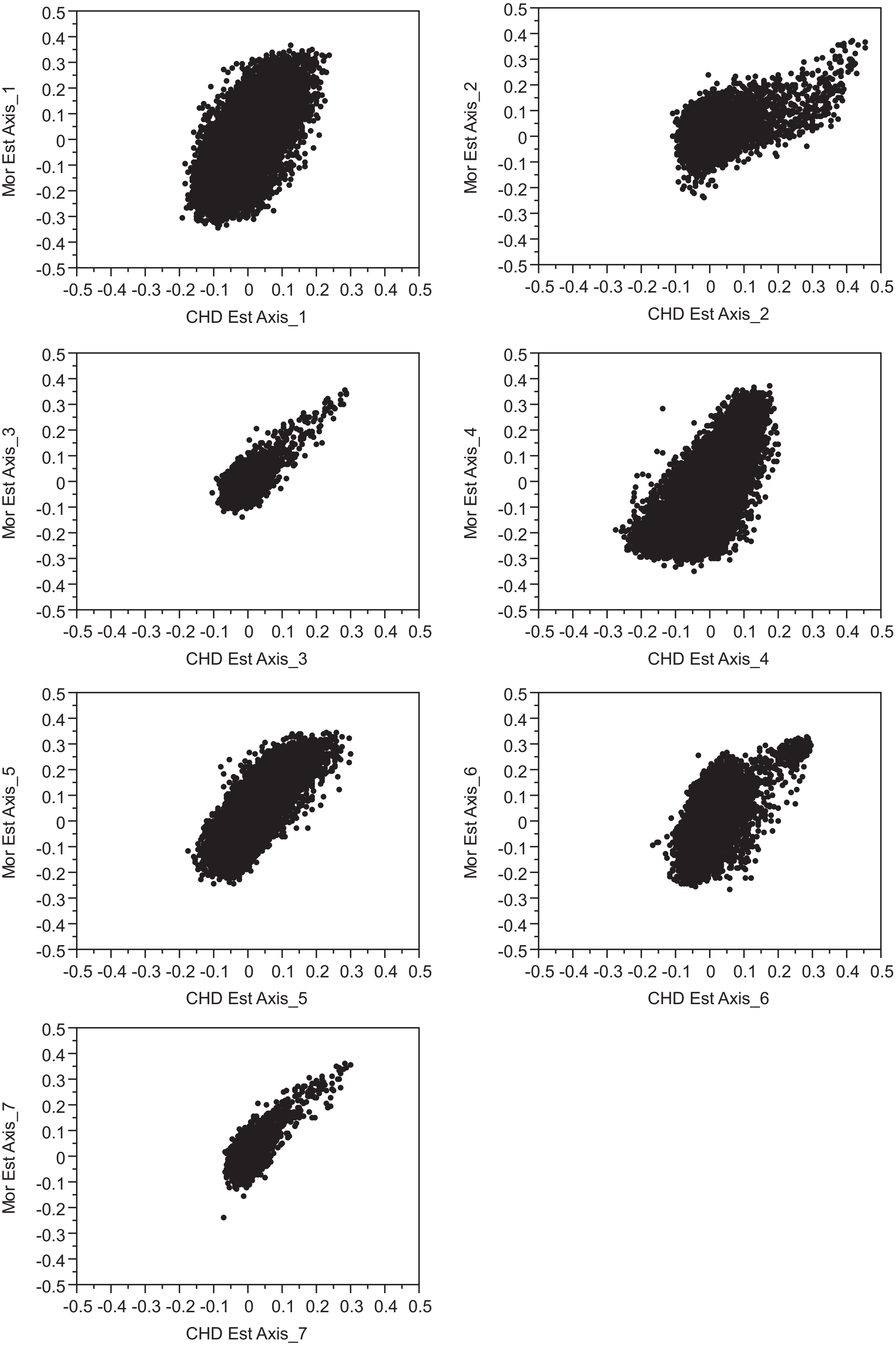

Supplementary Figure S3C   Preininger et al, 2012

Axis 1

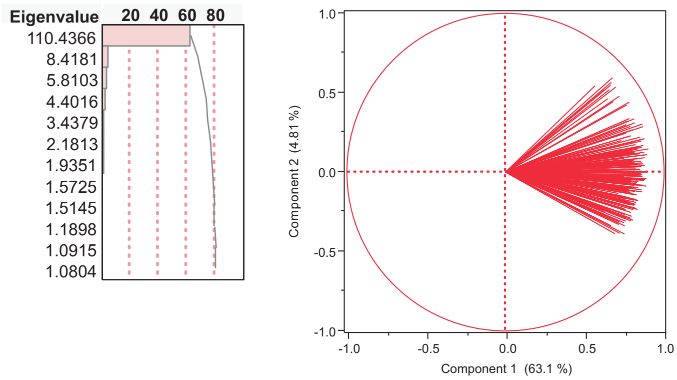

Axis 2

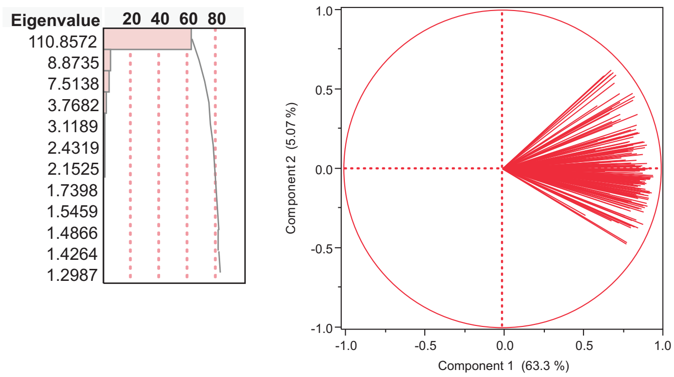

Axis 3

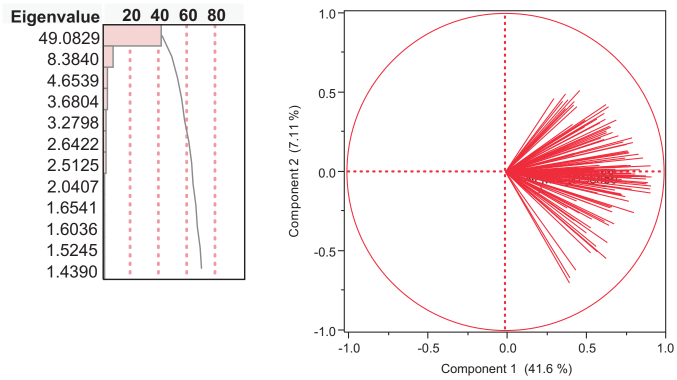

Axis 4

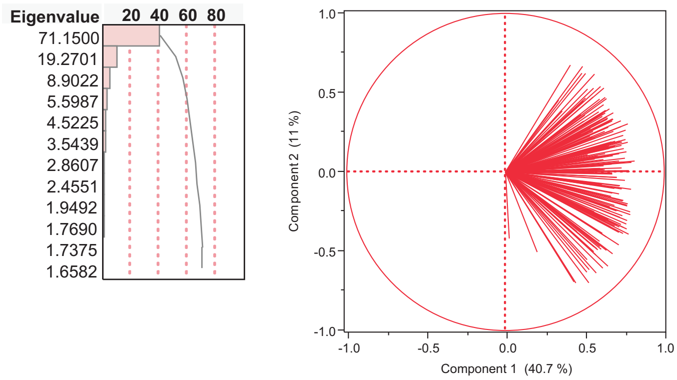

Axis 5

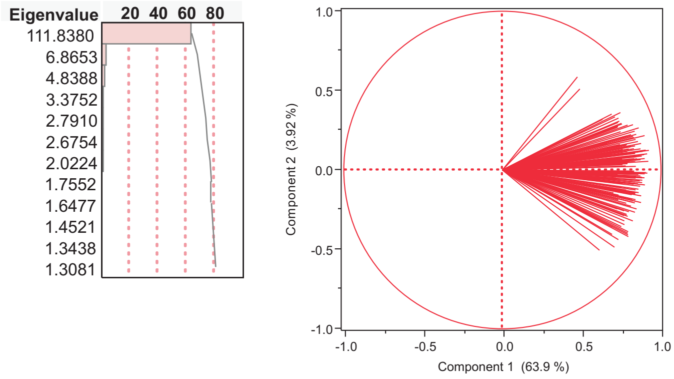

Axis 6

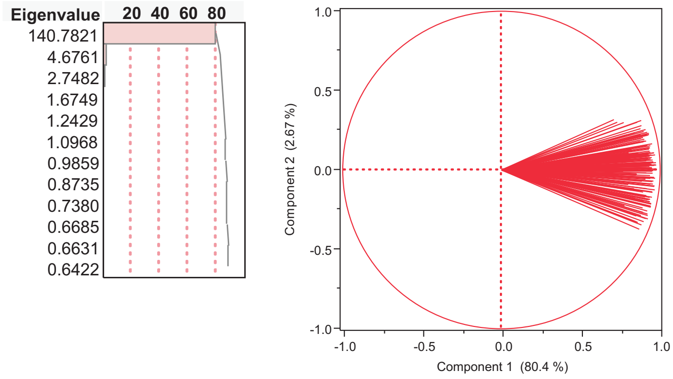

Axis 7

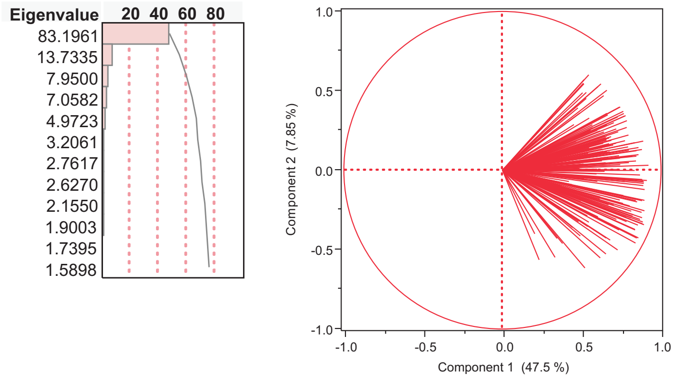

Module 3.5

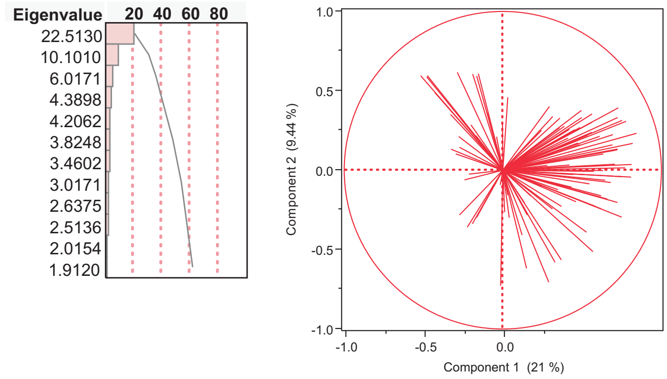

Supplement: Figure S3 — Blood Informative Axis scores. (A) Each plot shows the PC1 loadings of the 10 Bit transcripts in the CHDWB study on the right, and the individual PC1 and PC2 scores on the left. The same result for a typical random set of 10 probes is included as well. Panel J shows a histogram of the percent variance explained by PC1 for 100 random sets of 10 transcripts, relative to that observed for each of the BIT Axes, which are unambiguous outliers. (B) Pairwise comparison of the multiple regression coefficient for each transcript in the CHDWB/Atlanta and Morocco studies for each Axis, showing transcriptome-wide similarity for Axes 1, 3, 4, 5 and 7, whereas a subset of transcripts are clearly more strongly associated with Axes 2 and 6. (C) Principal component analysis of the 175 probes most strongly associated with each Axis, compared with a similar plot for a typical Chaussabel module, 3.5. In each case, the histogram of eigenvalues to the left shows how PC1 (the Axis score) dominates the covariance. The amount of variation explained by Axes and Modules in each study is compared in Table S4. (PDF) [file pgen.1003362.s007.pdf]
