# Supplementary material for: Blood-Informative Transcripts Define Nine Common Axes of Peripheral Blood Gene Expression
Source: PLoS Genet. 2013 Mar 14;9(3):e1003362. doi: 10.1371/journal.pgen.1003362 (PMC3597511; doi:10.1371/journal.pgen.1003362)

Supplementary Figure S8. Preininger et al, 2012

A.

Variance Explained by IIT			
PC1	LCL	PB	Pcorr
Axis 1	61.3	63.8	0.05
Axis 2	18.0	67.6	0.14
Axis 3	43.6	63.5	0.88
Axis 4	84.0	84.3	0.05
Axis 5	44.7	77.0	0.05
Axis 6	91.1	90.0	0.88
Axis 7	69.2	74.3	0.01

B.

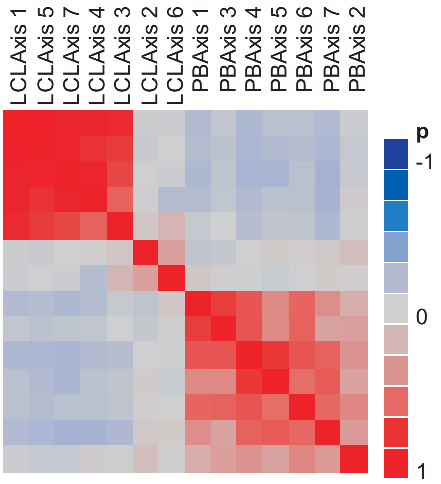

C.

Twin-Twin Correlations				
R	LCL		PB	
Axis 1	0.61	***	0.38	**
Axis 2	0.28	ns	0.34	*
Axis 3	0.35	ns	0.61	****
Axis 4	0.55	**	0.53	**
Axis 5	0.71	****	0.42	*
Axis 6	0.41	**	0.50	***
Axis 7	0.62	***	0.35	*
* p<.05	**p<.01	*** p<.0001	**** p<.00001	

D.

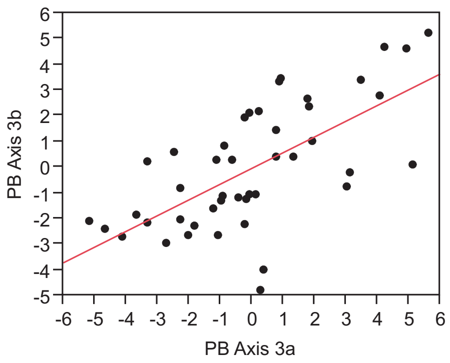

Supplement: Figure S8 — Heritability and differentiation of LCL and Peripheral Blood (PB) in the Brisbane twin study (18). PC1 scores for the 10 BIT per axis were computed for each dataset and are provided in Dataset S2. The percent variation of these BIT explained by PC1 is indicated in panel A, along with the significance (P-value) of the correlation between individual scores for LCL and PB. Six of the first seven BIT are also observed in lymphoblast cell lines (LCL), the exception being Axis 2. Nevertheless, there is no correlation between the axis scores for LCL and PB, as indicated in the heat map in panel B. Also, each of Axes 1,3,4,5 and 7 are highly correlated in LCL suggesting that they represent a single shared covariance structure. These results suggest that the whole blood profiles may arise by summation of contributions of different blood cell types. Although the LCL axes are different from PB ones, they also show significant heritability since the twin-twin phenotypic correlations are high for five of the axes as indicated in panel C. These were computed on the PC1 scores for the respective Axes, after removal of 2 outliers (and their twin partners) who are more than two standard deviations from the mean for multiple axes, indicated in italics on Dataset S2. The strongest twin-twin correlation is shown in panel D. These results imply that genetic factors contribute to the establishment of the shared covariance in individual cell types, and in peripheral blood as a whole. (PDF) [file pgen.1003362.s012.pdf]
