# Supplementary material for: Blood-Informative Transcripts Define Nine Common Axes of Peripheral Blood Gene Expression
Source: PLoS Genet. 2013 Mar 14;9(3):e1003362. doi: 10.1371/journal.pgen.1003362 (PMC3597511; doi:10.1371/journal.pgen.1003362)
Supplement: Table S1 — List of Blood Informative Transcripts including the Probe ID for the Illumina Human_HT12 bead chips. (DOCX) [file pgen.1003362.s013.docx]

**Supplementary Table 1**. List of Blood-Informative Transcripts

| **Axis** | **Probe** | **Gene** |
| --- | --- | --- |
| Axis 1 | ILMN_2309245 | BIN1 |
|  | ILMN_2098616 | C5ORF39 |
|  | ILMN_1700628 | DDX24 |
|  | ILMN_1703565 | GLTSCR2 |
|  | ILMN_1733696 | IMP3 |
|  | ILMN_1708151 | LAGE3 |
|  | ILMN_2183687 | LIME1 |
|  | ILMN_1700306 | OCIAD2 |
|  | ILMN_1657204 | SAE1 |
|  | ILMN_2369785 | SNRPD2 |
|  |  |  |
| Axis 2 | ILMN_1814397 | EPB42 |
|  | ILMN_1729487 | GMPR |
|  | ILMN_1759155 | IFIT1L |
|  | ILMN_1811927 | OR2W3 |
|  | ILMN_1784678 | PBX1 |
|  | ILMN_1680652 | SELENBP1 |
|  | ILMN_1772809 | SLC4A1 |
|  | ILMN_1704446 | SLC6A10P |
|  | ILMN_1766165 | SNCA |
|  | ILMN_1807919 | TNS1 |
|  |  |  |
| Axis 3 | ILMN_1775235 | AFF3 |
|  | ILMN_1668277 | BLK |
|  | ILMN_1782704 | CD19 |
|  | ILMN_1723004 | CD72 |
|  | ILMN_1734878 | CD79A |
|  | ILMN_1778681 | EBF1 |
|  | ILMN_1664063 | FAM129C |
|  | ILMN_1691071 | FCRLA |
|  | ILMN_1811049 | POU2AF1 |
|  | ILMN_1700147 | VPREB3 |
|  |  |  |
| Axis 4 | ILMN_2357272 | BCLAF1 |
|  | ILMN_2374293 | DYRK1A |
|  | ILMN_2378048 | HNRPK |
|  | ILMN_1792997 | NPTN |
|  | ILMN_1681845 | PAPD4 |
|  | ILMN_1700834 | SLK |
|  | ILMN_2312275 | SRP54 |
|  | ILMN_1682316 | TRIM33 |
|  | ILMN_1668417 | WASPIP |
|  | ILMN_1795228 | ZFAND5 |
|  |  |  |
| Axis 5 | ILMN_1662524 | IL8RA |
|  | ILMN_1689836 | C5AR1 |
|  | ILMN_1666049 | NUP214 |
|  | ILMN_1715068 | AQP9 |
|  | ILMN_1808047 | PHC2 |
|  | ILMN_2372974 | SIRPA |
|  | ILMN_2368292 | TSEN34 |
|  | ILMN_1722218 | MBOAT7 |
|  | ILMN_1791771 | HCK |
|  | ILMN_1778723 | AMICA1 |
|  |  |  |
| Axis 6 | ILMN_1680279 | USP49 |
|  | ILMN_1692145 | ZNF14 |
|  | ILMN_2054554 | DTWD2 |
|  | ILMN_2075794 | NLRP8 |
|  | ILMN_2106658 | BLZF1 |
|  | ILMN_2162367 | DMC1 |
|  | ILMN_2222101 | N4BP2 |
|  | ILMN_2313889 | ZNF682 |
|  | ILMN_2330495 | OCIAD1 |
|  | ILMN_2407851 | IL17RD |
|  |  |  |
| Axis 7 | ILMN_1739428 | IFIT2 |
|  | ILMN_1729749 | HERC5 |
|  | ILMN_1657871 | RSAD2 |
|  | ILMN_2388547 | EPSTI1 |
|  | ILMN_1745397 | OAS3 |
|  | ILMN_2349061 | IRF7 |
|  | ILMN_1799467 | SAMD9L |
|  | ILMN_1670305 | SERPING1 |
|  | ILMN_1662358 | MX1 |
|  | ILMN_1797001 | DDX58 |
|  |  |  |
| Axis 8 | ILMN_2066348 | HERPUD2 |
|  | ILMN_1783852 | CD164 |
|  | ILMN_2379788 | HIF1A |
|  | ILMN_2179397 | TATDN1 |
|  | ILMN_1713752 | SERINC3 |
|  | ILMN_1751816 | MCTS1 |
|  | ILMN_2044226 | PPP3CA |
|  | ILMN_1742813 | TMEM167A |
|  | ILMN_1739583 | ROCK1 |
|  | ILMN_2389844 | SP3 |
|  |  |  |
| Axis 9 | ILMN_1674780 | SF3B1 |
|  | ILMN_2272074 | TROVE2 |
|  | ILMN_1748797 | GRB2 |
|  | ILMN_2340217 | PTPRC |
|  | ILMN_1804148 | TMED4 |
|  | ILMN_1801984 | VHL |
|  | ILMN_1653652 | PTPRC |
|  | ILMN_1698323 | PLEKHB2 |
|  | ILMN_2291954 | SLA |
|  | ILMN_1756806 | MCL1 |
