# Supplementary material for: Blood-Informative Transcripts Define Nine Common Axes of Peripheral Blood Gene Expression
Source: PLoS Genet. 2013 Mar 14;9(3):e1003362. doi: 10.1371/journal.pgen.1003362 (PMC3597511; doi:10.1371/journal.pgen.1003362)
Supplement: Table S2 — Percent variance explained by PC1 for the 10 Blood Informative Transcripts for each Axis, in each of the 7 studies, showing high replication of their co-regulation. (DOCX) [file pgen.1003362.s014.docx]

**Supplementary Table S2**. Replication of Blood Informative Axes across 7 studies

|  | CHDWB | Morocco | BNE RC | BNE Twin | Celiac | TB | DILGOM |
| --- | --- | --- | --- | --- | --- | --- | --- |
| Axis 1 | 71.4 | 66.0 | 52.9 | 63.8 | 81.9 | 68.6 | 62.7 |
| Axis 2 | 84.4 | 73.6 | 59.4 | 69.3 | 73.8 | 76.2 | 80.1 |
| Axis 3 | 76.6 | 72.0 | 68.5 | 63.5 | 72.6 | 76.1 | 70.2 |
| Axis 4 | 53.4 | 69.4 | 54.7 | 84.3 | 92.3 | 35.3 | 46.0 |
| Axis 5 | 75.6 | 76.5 | 71.4 | 82.8 | 73.1 | 72.8 | 70.5 |
| Axis 6 | 92.9 | 93.4 | 84.7 | 90.0 | 98.3 | 93.1 | 91.5 |
| Axis 7 | 82.5 | 77.2 | 82.5 | 68.7 | 80.7 | 82.8 | 80.4 |
| Axis 8 | 58.9 | 69.3 | 51.7 | 85.7 | 85.2 | 64.9 | 44.4 |
| Axis 9 | 53.3 | 78.6 | 48.3 | 60.8 | 75.0 | 82.8 | 52.9 |

Values are the percent variance of 10 BIT abundance explained by each Axis PC1 in the Atlanta CHDWB, Morocco (13), Red Cross donors in Brisbane Australia (14), an independent Twin Study in Brisbane (18), a Celiac disease study (16), a Tuberculosis study in London and South Africa (15), and the DILGOM Finnrisk study (17). Not all probes are present in all studies, notably (16) averages just 7 probes per BIT.
