# Supplementary material for: Blood-Informative Transcripts Define Nine Common Axes of Peripheral Blood Gene Expression
Source: PLoS Genet. 2013 Mar 14;9(3):e1003362. doi: 10.1371/journal.pgen.1003362 (PMC3597511; doi:10.1371/journal.pgen.1003362)
Supplement: Table S3 — List of 9 studies referred to in the paper, showing the Location of the population, number of samples, source of blood RNA, GEO or ArrayExpress accession number, and reference in this paper (CHDWB is reported for the first time). (DOCX) [file pgen.1003362.s015.docx]

**Supplementary Table S3.** List of Studies

| Name | Location | N | Blood source | Accession | Ref |
| --- | --- | --- | --- | --- | --- |
| CHDWB | Atlanta, GA USA | 189 | Tempus (whole blood) | GEO GSE35846 |  |
| MOROCCO | Southern Morocco | 208 | Leukolock (leukocytes) | GEO GSE17065 | 13 |
| DILGOM | Finland | 518 | Paxgene (whole blood) | AE E-TABM-1036 | 17 |
| BNE-RC | Brisbane, Australia | 100 | Leukolock (leukocytes) | GEO GSE21311 | 14 |
| BNE Twin | Brisbane, Australia | 94 | Paxgene (whole blood) | GEO GSE33321 | 18 |
| TB | London | 96 | Tempus (whole blood) | GEO GSE19491 | 15 |
| TB | South Africa | 51 | Tempus (whole blood) | GEO GSE19442 | 15 |
| CELIAC | United Kingdom | 132 | Paxgene (whole blood) | GEO GSE11501 | 16 |
| Chaussabel | Dallas, TX USA | 304 | Ficoll gradient (PBMC) | GEO GSE11907 | 12 |
