# Supplementary material for: Blood-Informative Transcripts Define Nine Common Axes of Peripheral Blood Gene Expression
Source: PLoS Genet. 2013 Mar 14;9(3):e1003362. doi: 10.1371/journal.pgen.1003362 (PMC3597511; doi:10.1371/journal.pgen.1003362)
Supplement: Table S4 — Percent variance explained by PC1 for the entire set of probes annotated to the Chaussabel module genes (PVE_CHD and PVE_MOR refer to CHDWB Atlanta, and Morocco studies respectively), also showing the number of probes and genes, the Axis each module associates with, and the average for each measure. The bottom rows show the same values for the 175 most strongly associated axis genes for the first 7 axes from Dataset S1, and their averages. (DOCX) [file pgen.1003362.s016.docx]

**Supplementary Table 4**. Percent Variance Explained by PC1 for Modules and Axes

| Module | Probes | Genes | PVE_CHD | PVE_MOR | Axis |
| --- | --- | --- | --- | --- | --- |
| M1.1 | 21 | 17 | 31.2 | 31.1 | A3 |
| M1.2 | 109 | 79 | 39.8 | 36.0 | A2 |
| M1.3 | 51 | 38 | 43.4 | 32.5 | A3 |
| M1.4 | 119 | 80 | 14.6 | 18.9 | A4 |
| M1.5 | 131 | 94 | 32.8 | 30.2 | A5 |
| M1.6 | 122 | 85 | 18.2 | 18.8 | - |
| M1.7 | 97 | 56 | 35.4 | 28.9 | A1 |
| M1.8 | 159 | 110 | 17.9 | 18.2 | - |
| M2.1 | 89 | 60 | 42.3 | 33.3 | A1 |
| M2.2 | 38 | 32 | 36.8 | 29.2 | A2 |
| M2.3 | 98 | 71 | 54.2 | 24.5 | A2 |
| M2.4 | 109 | 67 | 36.9 | 24.6 | A1 |
| M2.5 | 107 | 79 | 15.2 | 25.5 | A2 |
| M2.6 | 167 | 105 | 36.3 | 34.6 | A5 |
| M2.7 | 66 | 40 | 13.5 | 19.6 | A4 |
| M2.8 | 119 | 83 | 36.0 | 28.0 | A1 |
| M2.9 | 195 | 114 | 20.4 | 23.0 | A4 |
| M2.10 | 95 | 55 | 29.0 | 23.7 | - |
| M2.11 | 179 | 108 | 16.7 | 20.4 | A4 |
| M3.1 | 108 | 73 | 47.3 | 39.3 | A7 |
| M3.2 | 346 | 222 | 20.3 | 19.5 | A5 |
| M3.3 | 370 | 166 | 27.6 | 23.9 | A5 |
| M3.4 | 432 | 187 | 17.6 | 21.1 | A4 |
| M3.5 | 107 | 72 | 21.0 | 20.2 | A5 |
| M3.6 | 353 | 166 | 17.2 | 18.1 | A4 |
| M3.7 | 394 | 210 | 22.2 | 18.3 | - |
| M3.8 | 332 | 133 | 16.0 | 20.1 | A1 |
| M3.9 | 372 | 147 | 16.3 | 19.3 | A4 |
| **Avg** | **175** | **98** | **27.7** | **25.0** |  |
|  |  |  |  |  |  |
| Axis 1 | 175 | 165 | 63.1 | 46.5 |  |
| Axis 2 | 175 | 157 | 63.3 | 34.6 |  |
| Axis 3 | 118 | 99 | 41.6 | 32.4 |  |
| Axis 4 | 175 | 158 | 40.7 | 60.2 |  |
| Axis 5 | 175 | 150 | 63.9 | 61.5 |  |
| Axis 6 | 175 | 171 | 80.4 | 74.4 |  |
| Axis 7 | 175 | 134 | 47.5 | 42.1 |  |
| **Avg** | **175** | **148** | **57.2** | **50.2** |  |
