# Supplementary material for: Blood-Informative Transcripts Define Nine Common Axes of Peripheral Blood Gene Expression
Source: PLoS Genet. 2013 Mar 14;9(3):e1003362. doi: 10.1371/journal.pgen.1003362 (PMC3597511; doi:10.1371/journal.pgen.1003362)
Supplement: Table S5 — Correlation of Overall transcriptome PCA with Meta-Modules PC1 in Atlanta and Morocco. The values are the correlation coefficients between the first Principal Component for each of the indicated Meta-Modules and for Axis 6, and the first 5 Principal Components (Prin1-5) of the entire gene expression dataset for both the CHDWB Atlanta, and Morocco, studies separately. Bold values are consistent between the two studies, noting that Prin3 and Prin4 negatively correlate with Axis 6 respectively. (DOCX) [file pgen.1003362.s017.docx]

**Supplementary Table 5**. Meta-Module-Standard PCA correlations

|  |  |  | Atlanta |  |  |  |  |  | Morocco |  |  |
| --- | --- | --- | --- | --- | --- | --- | --- | --- | --- | --- | --- |
|  | Prin1 | Prin2 | Prin3 | Prin4 | Prin5 |  | Prin1 | Prin2 | Prin3 | Prin4 | Prin5 |
| MM1 | 0.74 | **-0.48** | -0.13 | 0.23 | 0.12 |  | -0.09 | **-0.90** | 0.29 | 0.02 | -0.04 |
| MM2 | **-0.76** | 0.23 | -0.10 | 0.49 | -0.06 |  | **-0.75** | 0.02 | -0.06 | -0.23 | 0.11 |
| MM3 | 0.06 | -0.32 | -0.16 | 0.21 | -0.03 |  | 0.06 | -0.27 | 0.26 | -0.13 | 0.03 |
| MM4 | **0.92** | 0.27 | 0.03 | 0.03 | 0.03 |  | **0.78** | 0.54 | 0.16 | -0.05 | -0.06 |
| MM5 | 0.28 | 0.90 | 0.25 | -0.10 | 0.03 |  | -0.15 | 0.55 | -0.66 | 0.36 | 0.13 |
| MM6 | 0.03 | 0.38 | -0.1 | -0.12 | -0.03 |  | -0.02 | 0.19 | -0.42 | 0.15 | 0.01 |
| Axis 6 | -0.01 | 0.39 | **-0.80** | -0.02 | -0.19 |  | -0.18 | -0.4 | -0.43 | **-0.61** | -0.14 |
